# The genome sequence and demographic history of *Przewalskia tangutica* (Solanaceae), an endangered alpine plant on the Qinghai–Tibet Plateau

**DOI:** 10.1093/dnares/dsad005

**Published:** 2023-04-04

**Authors:** Ying Wu, Jiao Yang, Yongzhi Yang, Jianquan Liu

**Affiliations:** State Key Laboratory of Herbage Improvement and Grassland Agro-Ecosystem, College of Ecology, Lanzhou University, Lanzhou, China; State Key Laboratory of Herbage Improvement and Grassland Agro-Ecosystem, College of Ecology, Lanzhou University, Lanzhou, China; State Key Laboratory of Herbage Improvement and Grassland Agro-Ecosystem, College of Ecology, Lanzhou University, Lanzhou, China; State Key Laboratory of Herbage Improvement and Grassland Agro-Ecosystem, College of Ecology, Lanzhou University, Lanzhou, China

**Keywords:** *Przewalskia tangutica*, selfing, demographic history, hybridization, Qinghai–Tibet Plateau

## Abstract

To adapt to high-altitude habitats, many alpine plants develop self-compatible breeding systems from outcrossing. The genetic bases for this shift and the resulting demographic consequences remain largely unexplored. Here, we present a high-quality, chromosome-level genome assembly of the monotypic and endangered alpine perennial *Przewalskia tangutica* (Solanaceae) occurring on the Qinghai–Tibet Plateau (QTP). Our assembled genome is approximately 3 Gb, with a contig N50 size of 17 Mb, and we identified one lineage-specific whole-genome duplication. We found that the gametophytic self-incompatibility (GSI) syntenic locus to the other obligate outcrossing Solanaceae species was broken by the inserted the long terminal repeats, and changes in the flower-specific expression of the homologous genes, and the linked GSI genes in this species. Such changes may have led to its self-compatibility. We identified three deeply diverged lineages in the central distribution of this species, and the gene flow between them was weak but continuous. All three lineages diverged and decreased their population sizes since the largest glaciations occurred in the QTP approximately 720–500 thousand years ago. In addition, we identified one obvious hybrid population between two lineages, suggesting that genetic exchanges between and within lineages still occur. Our results provide insights into evolutionary adaptation through facultative self-pollination and demographic consequences of this alpine rare species in arid habitats.

## 1. Introduction

Many alpine plants on the Qinghai–Tibet Plateau (QTP), which is the highest (average elevation at above 4,000 m) and largest (ca. 2.5 million km2) plateau in the world, have developed special reproductive mechanisms to adapt to the arid habitat.^1,2^ The QTP is characterized by low temperature and strong UV radiation that result in a lack of sufficient effective pollinators.^1^ Therefore, many lineages develop self-compatible breeding systems in contrast to obligate outcrossing in the closely related species at lower altitudes. A few genomic analyses of such alpine plants have revealed genetic changes for this shift in breeding systems via functional loss of the obligate self-incompatibility (SI) genes.^3–6^ However, the demographic consequences of this facultative self-compatible system remain largely unknown based on population genomic analyses.

In this study, we generated a chromosome-level genome assembly for an alpine plant, *Przewalskia tangutica.* This is the only species in the monotypic genus of the tribe Hyoscyameae of the family Solanaceae and is an endangered plant, occurring in sandy and gritty grasslands at altitudes between 3,200 and 5,200 m in the central QTP.^7^ Its roots contain high concentrations of hyoscyamine and apoatropine, which are used as treatments for multiple diseases.^8^ Because of its medicinal importance, this species has been subjected to extensive collection, and the sizes of most populations have greatly decreased, and many have even been extinguished.^9^ Currently, *P. tangutica* is included in the Class I endangered Tibetan medicine directory.^7^ Although most Solanaceae species are obligate outcrossings via gametophytic self-incompatibility (GSI), this species is self-compatible with the dominant self-pollination.^10^ Many flowers have been pollinated underground through automatic self-pollination before the total plant grows out of buried soils.^11^ However, a few individuals flower above the ground and can be pollinated by insects according to field observations. It is likely that the self-incompatible system in this species may have broken, and the dominated self-pollination may have promoted intraspecific divergence of this endangered species. We also expected to find that rare outcrossings through pollinators may still persist within this species.

To test these hypotheses, we first characterized the genome sequence of *P. tangutica*. We then examined the genetic mutations at the collinear GSI locus of the family and the demographic history of this endangered species. We hope that these results will provide a better understanding of the evolution and adaptation of alpine plants and the Solanaceae family in the future.

## 2. Materials and methods

### 2.1. Plant materials, genomic DNA, and sequencing

Fresh leaves of *P. tangutica* were collected from Maduo County, Qinghai Province, China at an altitude of 4100 m (98°29ʹE, 34°35ʹN). We prepared high-molecular-weight genomic DNA from these leaves using a QIAGEN Genomic Kit. In addition, for transcriptome sequencing, we sampled several organs and tissues from *P. tangutica*, including the leaf, sepal, flower, and calyx from the three stages of fruit development. Three biological replicates from different individuals were used for gene-expression analysis. High-quality *P. tangutica* genomic DNA fragments (>20 kb) were selected using the Blue-pippin system (Saga Science) and used to construct the long-read libraries on the PromethION platform (https://nanoporetech.com). The libraries were sequenced using GRIDION X5 (v9.4.1; Oxford Nanopore Technologies) with 22 nanopore ﬂow cells and the SQK-LSK108 sequencing kit. Base calling of the raw nanopore reads was performed using the Oxford Nanopore base caller GUPPY v3.2.2 with default parameters. Nanopore reads with mean quality scores ≥7 (q7) were retained for the following genome assembly.

For the Illumina sequencing, one library with an insert size of 350 bp was constructed and sequenced using the Illumina Hiseq X Ten platform (Illumina, San Diego, CA, USA), representing the short-read sequencing library. Paired-end raw reads were trimmed using TRIMMOMATIC v.0.39^12^ to remove adaptors, reads with >3% N, and low-quality reads. The filtered clean data were used for *k*-mer analysis and error correction. For Hi-C sequencing, young leaves from the same plant were fixed in 1% formaldehyde for cross-linking. Cells were lysed using a Dounce homogenizer and digested using HindIII restriction enzyme. Complexes containing the biotin-labelled ligation products were purified and sheared, and the biotinylated Hi-C ligation products were removed and used to construct Illumina sequencing libraries.^13^ The resulting libraries were sequenced on an Illumina HiSeq X Ten platform to establish the chromosome-level genome assembly.

### 2.2. Genome assembly and chromosome construction

Before genome assembly, *k*-mer frequency distribution analysis was applied to estimate heterozygosity and genome size (genome size = total number of *k*-mers/peak depth).^14^ A total of 145.26-Gb Illumina short reads were used to determine the total number of *k*-mers with a length of 17 bp by Jellyfish.^15^*De novo* assembly of *P. tangutica* of the filtered Nanopore reads was performed using the NextDenovo v2.0-beta.1 assembler (parameters set: read_cutoff = 1k, seed_cutoff = 28k, blocksize = 8g) (https://github.com/Nextomics/NextDenovo.git). First, the NextCorrect module was applied to correct sequencing errors. Second, a preliminary assembly was generated based on the NextGraph module. Furthermore, Nanopore long reads and Illumina short reads were used for error correction based on Racon^16^ and Pilon^17^ software, respectively. Benchmarking Universal Single-Copy Orthologous gene analysis (BUSCO) with the 1,614 genes from Embryophyta_odb10 was used to further evaluate the completeness of the assembled genome.^18^ Then, the Hi-C paired-end reads were mapped to the draft assembled sequence using Bowtie 2^19^ to obtain unique mapped paired-end reads. By combining with the valid Hi-C data, the LACHESIS^20^ (ligating adjacent chromatin enables scaffolding *in situ*) *de novo* assembly pipeline was subsequently used to produce chromosome-level scaffolds. The linking results were then manually curated to correct mis-joins and mis-assemblies by visualizing the interaction heatmap using Juicebox (https://github.com/aidenlab/Juicebox). A heatmap of the interaction matrix of all pseudo-chromosomes was plotted with a resolution of 100 kb by HiCPlotter (https://github.com/kcakdemir/HiCPlotter).

### 2.3. Repeat and non-coding RNA annotation

The chromosome-level assembly of the *P. tangutica* genome was annotated using the following steps. For repeat annotation, both similarity-based predictions and *de novo* approaches were adopted. We first used RepeatMasker v4.0.5^21^ with Repbase TE library^22^ and RepeatProteinMasker^21^ with the TE protein database to search for homologous repeat sequences in the genome. Then, *de novo*-based identification was performed by RepeatModeler,^21^ and LTR_FINDER^23^ was used to predict the repeat element boundaries and family relationships from genome data. In addition, we used the program Tandem Repeats Finder v4.09^24^ (http://tandem.bu.edu/trf/trf.html, with the parameters ‘Match = 2, Mismatch = 7, Delta = 7, PM = 80, PI = 10, Minscore = 50, and MaxPeriod = 2000’) to search for tandem repeats. Finally, all repeat identification results from different software were integrated by a local Perl script, and the generated bed file was used to eliminate redundancy as the final repeat annotation.

The miRNAs and snRNAs were annotated by alignment to Rfam databases (release 9.1)^25^ using INFERNAL v1.1.2.^26^ The tRNA genes were identified using tRNAscan-SE software.^27^ rRNA fragments were predicted by alignment of the rRNA template sequences from the Rfam database based on BLASTN analysis (E-value of 1e–10).

### 2.4. Gene prediction and functional annotation

Three complementary methods were used to predict protein-coding genes: homology-based, *de novo*, and transcriptome-based predictions. In homology-based predictions, protein sequences of six different species (*Arabidopsis thaliana*, *Solanum tuberosum*, *Solanum lycopersicum*, *Capsicum annuum*, *Nicotiana tabacum*, *and Solanum pennellii*) were downloaded and aligned to the repeat-masked *P. tangutica* genome by TBLASTN with an *E*-value cut-off of 1e-5, and gene models were defined using GeMoMa.^28^ The aligned sequence and candidate genomic regions were corrected and optimized by GeneWise^29^ for further prediction of exact protein-coding gene structures. For *de novo* prediction, we extracted complete, multi-exon genes, then removed redundant high-identity genes (with an all-to-all identity cut-off of 70%), and finally randomly selected 3,000 full-length genes as the best candidate and low-identity gene models for training. Three *de novo* prediction programs (Augustus,^30^ Genscan,^31^ and GlimmerHMM^32^) were utilized with *P. tangutica* gene models for *de novo* prediction. Genes with coding sequences of less than 150 bp were discarded. For transcriptome-based predictions, all RNA-seq data of mixed samples (flower, leaf, stem, and root) were mapped to the *P. tangutica* genome using TopHat v.2.0.8 and Cufflinks v.2.1.1. In addition, Trinity was used to assemble the RNA-req data, and the assembled transcripts were then aligned to the assembled genome to carry out ORF prediction by PASA v2.1.0 pipeline.^33^ All predictions of gene models predicted by the abovementioned approaches were finally integrated using EVidenceModeler software (EVM; v1.1.1)^34^ to generate a consensus gene set.

Functional annotations of the predicted protein-coding genes were applied by BLAST (*E*-value of 1e–5) against publicly available protein databases, including NCBI non-redundant^35^ and Swiss-Prot^36^ protein databases. GO annotations were finished by Blast2GO pipeline v3.1.3.^37^ InterProScan v4.8^38^ and HMMER v3.1^39^ analyses were performed against the InterPro^40^ and Pfam databases, respectively. Additionally, the gene set was mapped to the KEGG^41^ pathway database to identify the corresponding function of each gene.

### 2.5. Genome evolution analysis

The protein-coding genes from 14 species, *P. tangutica*, *O. sativa*, *V. vinifera*, *C. canephora*, *M. guttata*, *Pe. axillaris*, *N. tabacum*, *I. nil*, *L. chinense*, *S. lycopersicum*, *Ph. floridana*, *S. tuberosum*, *C. annuum*, and *S. melongena*, were analysed to identify gene family groups. Orthologous gene groups were identified by running the OrthoMCL^42^ program. We retained the longest transcripts of each gene model to eliminate redundancy caused by alternative splicing variations. Orthogroups with only one gene copy per species (Single-copy orthogroups) were collected and aligned using Mafft v7.313^43^ with the globalpair G-INS-i strategy. The alignments of each single-copy orthogroups were concatenated into a super alignment. The super alignments were then filtered by Gblocks v.0.91b^44^ to remove gap regions. Subsequently, phylogenetic trees were constructed by RAxML v8.2.1151^45^ using the GTRGAMMA model, and we performed 1,000 bootstrap analyses to test the robustness of each branch. CAFE v3.1^46^ was used to identify expansions and contractions of gene families following divergence predicted by the phylogenetic tree with a probabilistic graphical model. Genes in significantly expanded families were then used for Gene Ontology enrichment analysis. The KOBAS^47^ software was also used to test the statistical enrichment of genes in KEGG pathways. Finally, the MCMCtree program in the PAML^48^ package was applied to infer the divergence time based on the constructed phylogenetic tree. The MCMCtree running parameters were as follows: burn-in: 10,000, sample number: 100,000, and sample frequency: 2. Calibration points were selected from publications and the TimeTree website (http://www.timetree.org) as normal priors to constrain the age of the nodes. Trees were visualized and edited using FigTree (http://tree.bio.ed.ac.uk/software/figtree/).

### 2.6. Genome synteny and whole-genome duplication

Syntenic blocks within five other representative plant species (*L. chinense*, *S. lycopersicum*, *Ph. floridana*, *C. annuum*, and *V. vinifera*) were identified using whole-genome duplication integrated analysis (WGDI), which contained an improved version of ColinearScan.^49,50^ We further filtered all tandem duplicated gene pairs for the following analysis as suggested by previous studies.^51^ Synonymous substitutions per synonymous site (*Ks*) between collinear genes were estimated using the yn00 program as implemented in the PAML package.^48^ Finally, we illustrated *Ks* distribution and the dotplots of orthologous blocks using WGDI toolkit.^50^ In each collinear block, the median *Ks* of homologous genes were used to classify the blocks generated by each duplication event. The *Ks* values were marked on a collinear block with different colours using the WGDI toolkit.

### 2.7. Estimation of insertion time of the LTR (long terminal repeat)-RTs

The software LTR-finder v1.07^23^ (https://github.com/xzhub/LTR_Finder/find/master/, with parameters: -w 2, -d 0, -l 100) was used for the *de novo* detection of LTR-RTs. The identification parameters were as follows. For LTR-harvest: -overlaps best, -seed 20, -minlenltr 100, -maxlenltr 2000, -mindistltr 3000, -maxdistltr 25000, -similar 85, -mintsd 4, -maxtsd 20, -motif tgca, -motifmis 1, -vic 60, -xdrop 5, -mat 2, -mis -2, -ins -3, -del -3. The two datasets were integrated to remove false positives using the LTR-retriever^52^ packages. The 5ʹ-LTR is usually identical to the 3ʹ-LTR at the time when a retrotransposon is inserted into the genome. All LTR sequences identified with complete 5ʹ-LTR and 3ʹ-LTR were used. Each of the 5ʹ-LTR flanking sequences and 3ʹ-flanking sequences was aligned by MUSCLE^53^ (v3.8.31, http://www.drive5.com/muscle, with default parameters) and the distance of the alignment sequences was calculated by the disMat (EMBOSS: v6.6.0.0, http://emboss.sourceforge.net/, with parameters-nucmethod). Evolutionary distances were converted into insertion times, assuming an equal neutral substitution rate of 1.33 × 10^−9^ per site per generation. The insertion time was calculated using the following formula: T = *K*/2r (divergence between LTRs/substitution per site per year). All LTR segments from the full-length LTR-RTs were used as queries to blast (*E*-value: 1e–6) against the genome sequences to identify homologous fragments. We also screened for solo-LTRs that did not overlap with any full-length LTR-RTs based on a previously established method.^54^

### 2.8. Identification of S-RNase and SLF gene sequences at the GSI locus

To identify candidate S-RNases involved in SI, 22 published RNase-T2 sequences and 73 SLF sequences from Solanaceae species^55^ were downloaded from NCBI (Supplementary Tables S22 and S23). The published sequences were used as a query to identify corresponding genes in the *P. tangutica* genome using BLAST. Via searches, we manually annotated the candidate homologous gene of the GSI locus. Conserved domains were identified using a combination of BLASTP and InterProScan v5.^56^ Sequences were aligned using ClustalW,^57^ and the alignment was used as the input to the MEGA6 maximum-likelihood phylogenetics analysis, using the bootstrap method with 1,000 iterations. By constructing a phylogenetic tree, we divided the RNase-T2 gene family into three subfamilies.^55^ All candidate proteins of the GSI locus were further confirmed by hmmsearch against the Pfam database. Then, genome sequences were loaded into MCScanX (Python version), a package from the JCVI utility libraries (https://github.com/tanghaibao/jcvi).^58^ The comparisons between gene pairs were performed using LAST (https://github.com/mcfrith/last-genome-alignments). After the removal of hits with low scores, the anchors from the LAST outputs were clustered into syntenic blocks. Synteny blocks between each pair of candidate species were performed with parameters ‘-a, -e 1e-5, -s 5’. The targeted S-RNase genes or neighbouring genes were chosen as seeds to search for synteny blocks of conserved evolution. Finally, microsynteny plots were generated using the “synteny” function with default parameters.

### 2.9. Transcriptome analysis

RNAs were extracted from four tissue types (leaf, sepal, flower, and calyx for the three stages of fruit development) of *P. tangutica* using an RNeasy Plus Mini Kit (QIAGEN). Then, mRNA isolation, fragmentation, and purification were performed using a TruSeq RNA Library Prep Kit v.2 (Illumina, USA). The raw sequencing data from three replicates of the different tissues were trimmed using TRIMMOMATIC v.0.39.^12^ Transcripts per kilobase of exon model per million reads mapped (TPM) values for RNA-Seq reads were calculated using HISAT2 v2.0.5 and CUFFLINKS v2.2.1.^59^ The gene heatmap figures were produced using TBtools^60^ with the Heatmap Illustrator function.

### 2.10. SNPs calling from genome resequencing individuals

A total of 30 individuals from six natural populations of *P. tangutica* were collected from its central distributions on the QTP. Paired-end libraries were constructed using the Illumina library preparation pipeline, and 840 Gb of reads with an average depth of ~20× were obtained from the Illumina HiSeq platform. All raw reads were filtered by the quality control software package fastp v0.20.0,^61^ and all clean reads were aligned to the reference genome of *P. tangutica* using bwa-mem v.0.7.17.^62^ Picard v2.23.4 (http://broadinstitute.github.io/picard/) was employed to add group information and remove PCR duplication in the sorted BAM files returned from samtools v1.9.^63^ The reads in insertion/deletion (Indel) intervals were realigned using RealignerTargetCreator and IndelRealigner modules in the Genome Analysis Toolkit (GATK) v3.8.1.^64^ Variant calling for each genome was carried out separately by GATK HaplotypeCaller to produce GVCF files, and all GVCFs were merged using the GATK Genotype GVCFs function. These SNPs were initially filtered using VariantFiltration (for SNPs: --filterExpression ‘QD<2.0, FS>60.0, MQ<40.0, MQRankSum<−12.5, ReadPosRankSum<−8.0’; for indels: --filterExpression ‘QD<2.0, FS>200.0, ReadPosRankSum<−20.0’). The SNPs were further filtered on custom Perl scripts to improve the quality of the SNPs by: removing SNP sites with two alleles and a quality score <20; marking SNP sites that have more than three times or less than one-third of the mean depth of individual sites as information missing; removing SNP sites containing a <20% missing ratio and those at or within 5 bp of Indels; and masking SNP sites located in repetitive regions. Vcftools v0.1.13^65^ with parameters ‘maf < 0.05, min-meanDP = 3, max-meanDP = 27, max-missing = 0.7, hwe = 0.001, and minGQ = 10’ was used to further reduce false positive SNPs, and the 64,148,143 high-quality SNPs finally obtained were used for downstream population genetic analyses.

### 2.11. PCA, population structure, and phylogenetic analysis

Based on the high-quality SNP data set, we first built a neighbour-joining tree with phylip v3.697^66^ under a distance matrix. The population structure was then inferred using Bayesian clustering in admixture v1.30^67^ with the number of ancestral clusters (K) from 2 to 6. Plink v1.90b6.7^68^ and smartpca from Eigensoft v7.2.1 (http://www.hsph.harvard.edu/alkes-price/software/) were run to perform principal component analysis (PCA). Genetic diversity (*π*) was calculated using 50-kb sliding windows in 25-kb steps for high-quality SNP datasets by Vcftools v0.1.13.^65^ The inbreeding coefficient (*F*_IS_) was estimated using the GCTA^69^ with SNP data. In addition, the selfing rate (*s*) was estimated as *s* = 2*F*_IS_/ (1+ *F*_IS_).^70^

### 2.12 . Demographic history

The pairwise sequentially Markovian coalescent (PSMC) model v.0.6.4-r49^71^ was used to infer the demographic history of *P. tangutica*. The analysis was performed using the following parameters: −N25 −t15 −r5 –p‘4 + 25 × 2 + 4+6’. A generation time of 3 years, given that the species is perennial, and a substitution rate (*μ*) of 7.8e–9 per site per year^72^ were used to scale the PSMC estimations. We inferred the demographic history of *P. tangutica* using a continuous-time coalescent simulator method in fastsimcoal v.2.7.^73^

## 3. Results

### 3.1. Genome sequence and assembly

**Figure 1.**
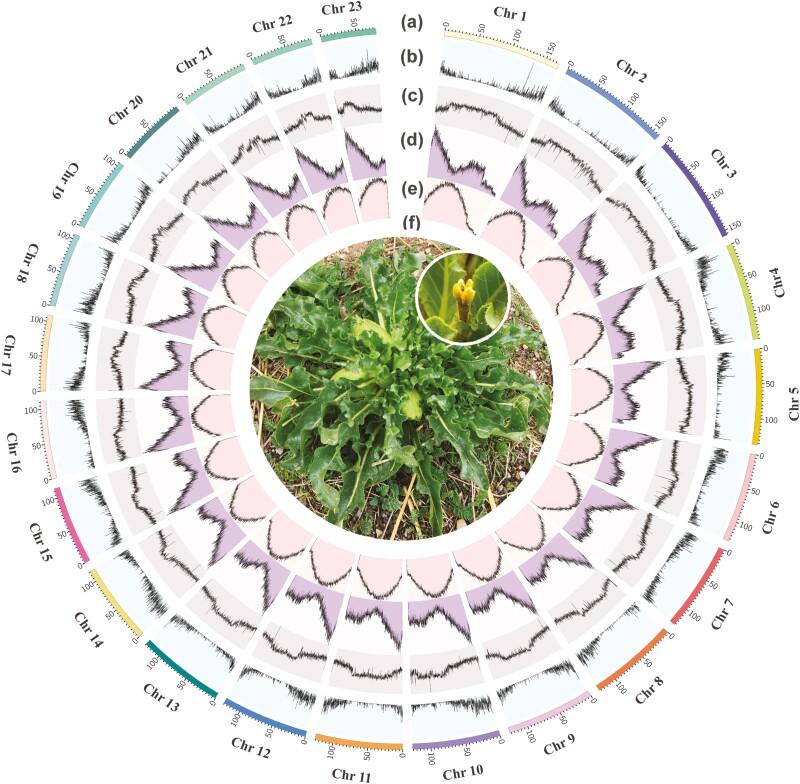
Characterization and features of the *P. tangutica* genome. (a) The genomic landscape of the 23 *P. tangutica* pseudo-chromosomes. All density information was counted in nonoverlapping 1-Mb windows. (b) Gene density, (c) guanine–cytosine content, (d) distribution of *Copia*-type retrotransposons, (e) distribution of *Gypsy*-type retrotransposons, and (f) one *P. tangutica* individual.

The genome size of *P. tangutica* was estimated to be 2.96 Gb with a low heterozygosity of 0.33%, as assessed by *k*-mer analysis based on 145.26-Gb clean short reads (Supplementary Fig. S1 and Supplementary Table S1). For genome sequencing, we obtained a total of 244.47 Gb of long-read sequencing data, which represents ~82.6-fold coverage of the estimated genome (Supplementary Table S1), using Oxford Nanopore Technologies. After *de novo* assembly via a hybrid approach, a contig-level *P. tangutica* assembly was generated with a length of ~3.03 Gb and contig N50 of 17.50 Mb, which is approximately equal to the estimated genome size in 1610 contigs (Table 1 and Supplementary Table S2). A total of 325 Gb of clean Hi-C reads (Supplementary Table S1) were generated, 92% of the assembled sequences were anchored onto 23 chromosomes (2*n* = 46) (Fig. 1, Supplementary Fig. S2, and Supplementary Table S3), and the total length of the assembly was 3.03G with a scaffold N50 of 125.83 Mb (Table 1). The longest chromosome was ~169.66 Mb, and the average length was 122.18 Mb (Supplementary Table S3). The quality of the *P. tangutica* genome assembly was further assessed by two methods. The alignment rate of all short reads to the genome was ~99.93%, and BUSCO analysis showed that the proportion of complete BUSCOs was 97.89% (Supplementary Table S4). All these results support the high quality of the assembled *P. tangutica* genome (Supplementary Table S4).

**Table 1.** Summary of *Przewalskia tangutica* genome assembly and annotation

Genomic feature	Nanopore assembly	Hi-C assembly
Assembled genome size (Mb)	3,028	3,028
Number of contigs/Scaffold	1,610	663
Contig N50 (Mb)/Scaffold N50 (Mb)	17.50	125.83
Longest contig (Mb)	73.15	169.66
GC content	40.28%	40.28%
BUSCO completeness of genome	97.89%	97.89%
Anchored to chromosome (Mb)		2,810
Masked repeat sequence length (Mb)		2,522
Percentage of repeat sequences (%)		83.27%
Number of predicted genes		50,828
Average gene length (bp)		4,372.30
Average CDS length (bp)		1,025.65
BUSCO completeness of gene set		92.69%

We further identified 50,828 protein-coding genes in the *P. tangutica* genome by multiple processes. The annotated gene number was greater than most genome-sequenced Solanaceae species (Supplementary Tables S5 and S6). These genes with an average length of 4.37 kb and an average exon number of 4.78 were similar to those of other Solanaceae species (Supplementary Fig. S3 and Supplementary Table S7). We also found that 92.69% of BUSCOs could be completely identified in our gene set (Table 1 and Supplementary Table S4), and 89.91% of genes could be successfully assigned to a functional annotation by five public database resources (Supplementary Table S8). Repetitive sequences were identified to represent 83.27% (2.52 Gb) in the *P. tangutica* genome (Supplementary Table S9). In addition, 18,391 non-coding RNA (ncRNA) genes were detected in the *P. tangutica* genome, including 644 microRNA (miRNA) genes with an average length of 153.99 bp, 3,080 transfer RNA (tRNA) genes, 2,887 ribosomal RNA (rRNA) genes, and 11,780 small nuclear RNA (snRNA) genes (Supplementary Table S10).

### 3.2. Evolution of the *P. tangutica* genome

**Figure 2.**
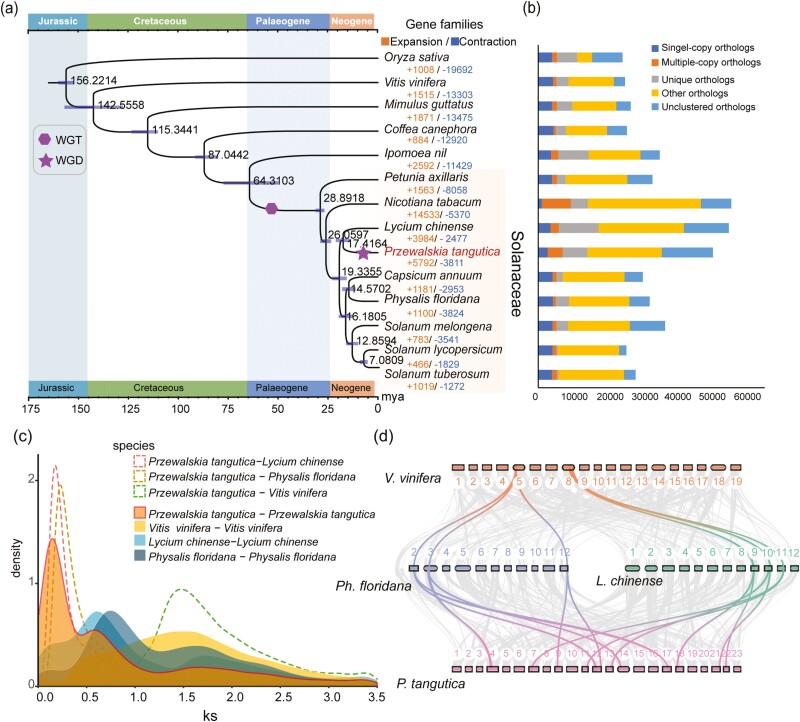
(a) Phylogenetic tree of the 14 plant species. The numbers denote divergence time of each node (Mya, million years ago), and the bars show the 95% confidence intervals of divergence times in millions of years. The timing of whole-genome duplication (WGD) and whole-genome triplication (WGT) events is superimposed on the tree. Gene family expansions and contractions are shown with numbers in different colors. (b) The distribution of single-copy, multiple-copy, unique, and other orthologs in the 14 plant species. (c) Distribution of synonymous substitution levels (*Ks*) of syntenic orthologous (dashed curves) and paralogous genes (solid curves). (d) Collinear relationship between *P. tangutica*, *Ph. floridana*, *L. chinense*, and *V. vinifera* chromosomes.

To reveal the evolutionary history of *P. tangutica* in the family Solanaceae, eight species from this family, four species from other major eudicot lineages, and one monocot (*O. sativa*) (as the outgroup) were selected for phylogenomic analyses (Supplementary Table S11). A total of 40,898 gene families were constructed by OrthoMCL, and 500 single-copy gene families were also retrieved (Supplementary Table S12). The highly supported species tree was obtained through maximum-likelihood analysis of the concatenated nucleotide sequences (Fig. 2a and Supplementary Fig. S4), and the phylogenetic relationship between and within the main clades agreed with previous studies.^74,75^ The resulting phylogeny indicated that *Petunia axillaris* and tobacco (*N. tabacum*) were successively sister to the seven other Solanaceae species, and their divergence times were estimated at 28.89–26.06 million years ago (Mya) by MCMCtree inference (Fig. 2a and Supplementary Fig. S5). *P. tangutica* was most closely related to wolfberry (*Lycium chinense*), and they split at ~17.42 Mya (Fig. 2a). *Physalis floridana* and pepper (*C. annuum*) clustered together with a divergence time of ~14.57 Mya, and they together diverged with the three *Solanum* species (*S. lycopersicum* [tomato], *S. tuberosum* [potato], and *S. melongena* [eggplant]) at ~16.18 Mya.

Significant expansion or contraction in the size of particular gene families is often associated with the adaptive divergence of closely related species.^76^ Based on the phylogenetic tree and the gene family data, we identified 5,792 expansion and 3,811 contraction gene families in *P. tangutica* (Fig. 2a). Gene Ontology (GO) terms and KEGG functional enrichment analysis of the expanded genes demonstrate that they were mainly associated with response to UV and other stresses, such as ‘cellular response to DNA damage stimulus’, ‘DNA repair’, ‘DNA repair and recombination proteins’, ‘response to stress’, and ‘environmental information processing’ (Supplementary Fig. S6 and Supplementary Tables S13 and S14). We further compared the transcription factor (TF) families between *P. tangutica* and other low-altitude Solanaceae species. A total of 25 TF families showed a significant expansion in *P. tangutica*, and most were found to be related to the abiotic stress response, including the *MYB*, *NAC*, *WRKY*, and *bHLH* gene families (Supplementary Table S15). All these expanded gene families may together enhance the stress responding ability and help *P. tangutica* adapt to arid QTP habitats.

### 3.3. *P. tangutica* experienced a lineage-specific whole-genome duplication event

Whole-genome duplication (WGD) is considered an important evolutionary force in plants and greatly contributes to their diverse environment adaptations.^4,77^ To investigate the WGD events during the evolution history of *P. tangutica*, the identified gene duplications were further classified into five types, among which WGD/segmental duplication was found to occupy 15% of the identified duplicate genes (Supplementary Fig. S7). A comparative genomic investigation was further performed among the six species of *P. tangutica*, *S. lycopersicum*, *C. annuum*, *L. chinense*, and *Ph. floridana* from Solanaceae, and *Vitis vinifera* as a reference, which only has the γ whole-genome triplication (WGT) event that is shared by all core eudicots (Fig. 2c and Supplementary Figs. S8 and S9).^78^ By constructing the distribution of synonymous substitutions per synonymous site (*Ks*) of homolog pairs from intragenomic analyses, we detected three *Ks* peaks in *P. tangutica* (Fig. 2c). The most ancient *Ks* peak represents the γ event that occurs in all six species with similar *Ks* boundaries (1.3–1.6). The middle peak represents the Solanaceae-common WGT event^75,79,80^ in which all five Solanaceae species showed a similar peak at ~0.6, whereas the most recent *Ks* peak (~0.13) was only found for *P. tangutica*, suggesting a recent species-specific polyploidization event (Fig. 2c and Supplementary Fig. S10). Combining *Ks* dot plots and synteny analysis, we found that the recent species-specific polyploidization event in *P. tangutica* was a whole-genome duplication (WGD) event (Supplementary Fig. S9). This was also confirmed by the syntenic depth ratio analyses. For each genomic region in *V. vinifera*, we typically found three matching regions in *Ph. floridana* and *L. chinense* with a similar level of divergence. We identified 2:1 syntenic depth ratios in the *P. tangutica–Ph. floridana* comparison and 2:1 syntenic depth ratios in the *P. tangutica–L. chinense* comparison, again supporting a recent WGD event that is unique to *P. tangutica* (Fig. 2d). We further estimated the time of the recent species-specific WGD event by assuming that the γ event occurred at 117 Mya^77^ with a *Ks* peak of 1.51 in *P. tangutica*. The recent *Ks* peak of 0.125 can be inferred at 9.69 Mya, which coincides with the extensive climate change in the QTP.^81^ During the late Miocene–early Pliocene (ca. 10–5 Mya), lineage-specific WGDs were also found in other plants.^81^ WGD has been hypothesized to buffer plants though episodes of climatic upheavals.^82^ The genes related to cold acclimation (Supplementary Fig. S11 and Supplementary Table S16), DNA damage repair (Supplementary Table S17), and UV radiation (Supplementary Fig. S12 and Supplementary Table S18) expanded due to WGD in *P. tangutica*. The species-specific WGD may have played an important role in the arid adaptation and high-altitude survival of this alpine plant.

### 3.4. Retrotransposon expansion in the *P. tangutica* genome

**Figure 3.**
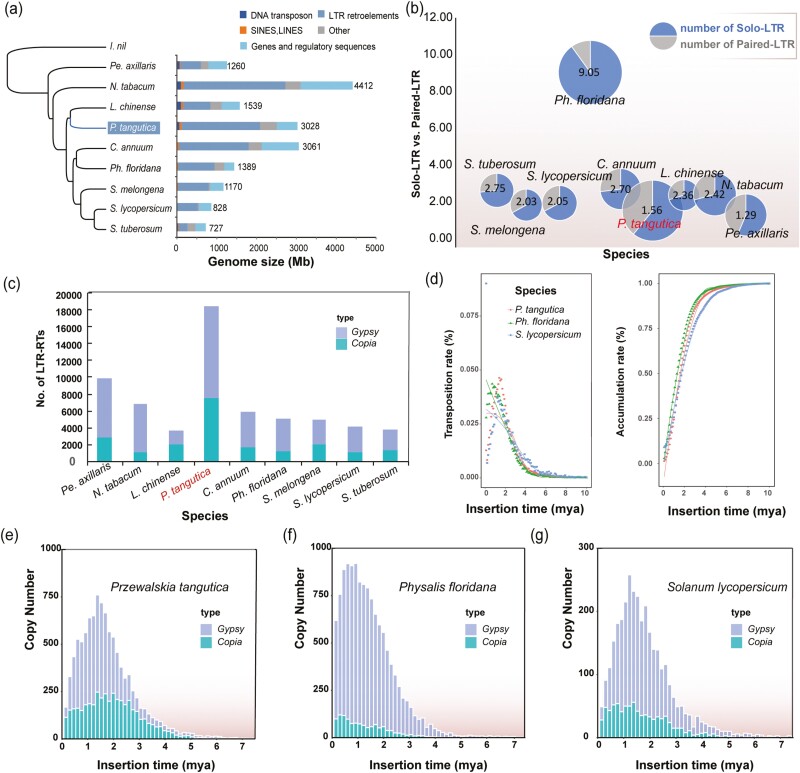
Genome size variation in the Solanaceae species. (a) Genome sizes and proportions of different types of genome organization in *P. tangutica*, *Ph. floridana*, *L. chinense*, *S. lycopersicum*, *S. tuberosum*, *S. melongena*, *C. annuum*, *N. tabacum*, and *Pe. axillaris*, using *I. nil* as the outgroup. (b) Comparison of solo- and paired-LTRs in Solanaceae species. (c) Number of full-length *Copia* and *Gypsy* elements in the nine genomes. (d) Transposition rates of LTR-RTs and accumulation rates of LTR-RTs in increments of 0.1 Mya over 10 Mya in *P. tangutica*, *Ph. Floridana*, and *S. lycopersicum*. (e–g) Distribution of insertion times of *Copia* and *Gypsy* elements in *P. tangutica*, *Ph. floridana*, and *S. lycopersicum*.

The genome size of *P. tangutica* is larger than those of most Solanaceae species (Fig. 3a). Except for WGD, we noticed that repetitive sequences constituted an important component of this enlarged *P. tangutica* genome (Supplementary Table S19). Because the genome size diversity in plants is primarily influenced by TEs, we compared the total content of repetitive sequences across the Solanaceae species (Fig. 3a). A total of 2.52 Gb of repetitive sequences occupied 83.27% of the entire genome of this species, and 82.97% were annotated as transposable elements (TEs) (Supplementary Table S19). Long terminal repeat retrotransposons (LTR) accounted for 65.11% of the total repetitive sequences and represented a majority, as found in other Solanaceae plants (Fig. 3a and Supplementary Table S19). In addition, Ty3/*Gypsy* LTR-retrotransposon families, at approximately 1,672.59 Mb (55.23% of the total genome), are 5.81-fold more abundant than Ty1/*Copia* with 287.66 Mb (9.50%) (Supplementary Table S19). The full-length LTR-RTs, which retain the paired LTRs at the two ends, may still possess a transposition ability and are the most abundant in the *P. tangutica* genome (Fig. 3c). The LTR-RT in *P. tangutica* was estimated to expand between 1 and 2 Mya (Fig. 3e), which is earlier than most Solanaceae species with smaller genome sizes (Figs. 3f and g and Supplementary Fig. S13).

DNA removal plays a major role in preventing TE proliferation-mediated genome expansion.^83,84^ Full-length LTR-RTs with a pair of identical direct repeats (paired-LTRs) are particularly favoured for DNA removal via unequal homologous recombination (HR) events because the two LTRs provide homologous sequences to initiate illegitimate recombination.^84,85^ Frequent HR-mediated DNA removal may result in a high abundance of solo-LTR remnants in the genome, which can be used as evidence to confirm the existence of an inherently efficient DNA removal mechanism. Thus, we compared the ratio of solo-LTR versus paired-LTR of *Copia* and *Gypsy* elements among Solanaceae species. We found that the ratio of solo-LTR/paired-LTR was considerably lower in *P. tangutica* (1.56; 28,714 solo-LTRs: 18,445 paired-LTRs) compared with other Solanaceae species, except for *Pe. axillaris* (1.29; 12766: 9889) (Fig. 3b and Supplementary Table S20). Solo-LTRs are thought to arise through excision-based DNA recombination between adjacent LTRs of the same element, which leads to the removal of repetitive sequences and genome downsizing.

We calculated the transposition rate as the net increase in the number of LTR-RTs within every 0.1 million years over a 10-million-year period (Fig. 3d). Within the last two million years, the transposition rate of *P. tangutica* has been relatively stable, whereas those of *Ph. floridana* and *S. lycopersicum* have continuously increased. The same tendency was also observed in terms of the accumulation rate of LTR-TRs (Fig. 3d). We further constructed phylogenetic trees of the domains in reverse transcriptase genes for both Ty1/*Copia* and Ty3/*Gypsy* superfamilies. The LTR-RTs in *P. tangutica* exhibited higher diversity and abundance, indicating greater expansion and divergence in *P. tangutica*. The *Copia* superfamily displayed a similar pattern, with four major clades consisting of elements from three species (Supplementary Fig. S14), suggesting a conserved evolution pattern of this superfamily.

### 3.5. GSI locus genes and self-compatibility of *P. tangutica*

**Figure 4.**
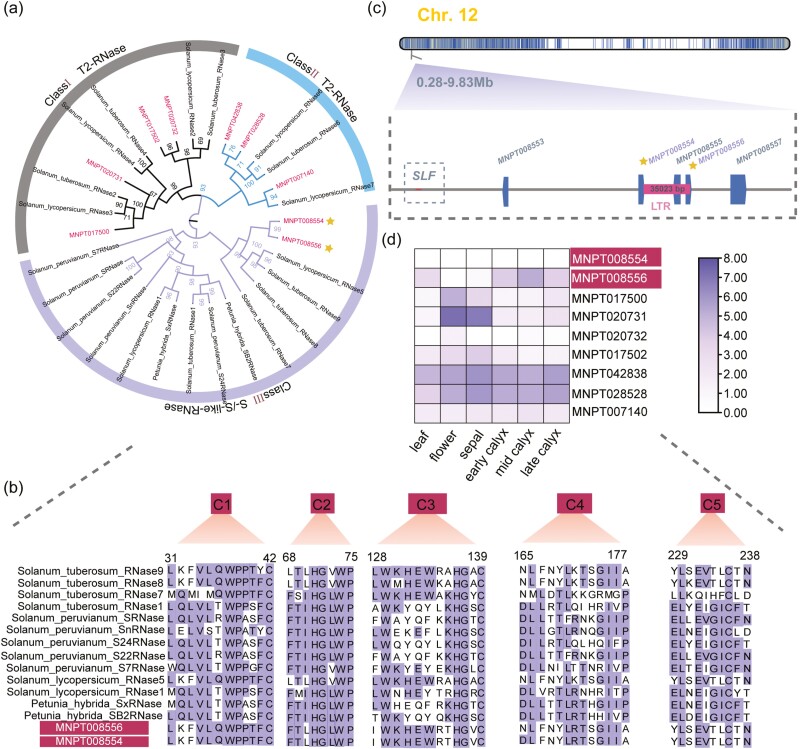
The RNase T2 genes from *P. tangutica* and other Solanaceae species. (a) Maximum-likelihood phylogenetic tree of the RNase T2 genes obtained from *P. tangutica* and RNase T2 genes from Solanaceae species with bootstrap values above 50%. (b) Amino acid sequence alignment of five conserved regions of the RNase T2 genes from the *P. tangutica* and diverse Solanaceae species. (c) Location of S-RNase genes of *P. tangutica* on chromosome 12 and a 35023-bp LTR insertion were identified across two RNase genes. The dotted square represents the deletion of the SLF gene. (d) Gene-expression levels in *P. tangutica*.

Self-fertilization is prevented by the S-RNase-mediated genes at the GSI locus in Solanaceae.^86^ Self-fertilization leads to decreased fitness of homozygous offspring and ensures reproduction in the absence of pollinators.^87,88^ The RNases-T2 and linked S-locus F-box (SLF) genes at the same locus are responsible for self-pollen recognition and rejection in the obligate outcrossing Solanaceae species.^86^ Nine RNase-T2 genes were identified in *P. tangutica*, and they could be divided into three subfamilies (Fig. 4a). The two genes (*MNPT008554* and *MNPT08556*) clustered together with the functionally confirmed S-RNase genes from the outcrossing Solanaceae species (Supplementary Table S22). However, amino acid sequence alignment revealed the low identities (48.70%) between these two RNase genes of *P. tangutica* and those of the outcrossing Solanaceae species (Fig. 4b). We found that a 35 kb LTR was inserted between these two genes in *P. tangutica* (Fig. 4c). In addition, we performed phylogenetic analysis of all SLF proteins encoded by those genes linked to the S-RNase genes in the other obligate outcrossing Solanaceae species (Supplementary Table S23). We identified a total of 10 SLF-like genes in the *P. tangutica* genome (Supplementary Fig. S15), but none were found to be linked to the two putative RNases genes (*MNPT008554* and *MNPT08556*) on chromosome12 (Fig. 4c and Supplementary Fig. S16), whereas identified SLF genes are successively linked at the GSI locus in the other outcrossing Solanaceae species.^55^

We finally identified the syntenic regions containing putative two S-RNase genes across the three obligate outcrossing Solanaceae species and *P. tangutica* via collinearity analysis. We revealed highly conserved blocks for two S-RNase genes across the four species, but no corresponding SLF gene was found in the upstream and downstream of the collinear region of *P. tangutica* (Supplementary Fig. S17). Therefore, our syntenic analyses revealed that the GSI locus of this species may have been degraded through the loss of the SLF gene and the LTR insertion in the putative syntenic S-RNase genes. In addition, the two RNases genes did not express specifically in flowers as other outcrossing species but expressed in other tissues, suggesting that they may have changed their original functions in GSI (Fig. 4d). Thus, we speculate that these changes may have been associated with the disrupted GSI and resulted in the self-compatibility and self-pollination in *P. tangutica.*

### 3.6. Population structure and demographic history

**Figure 5.**
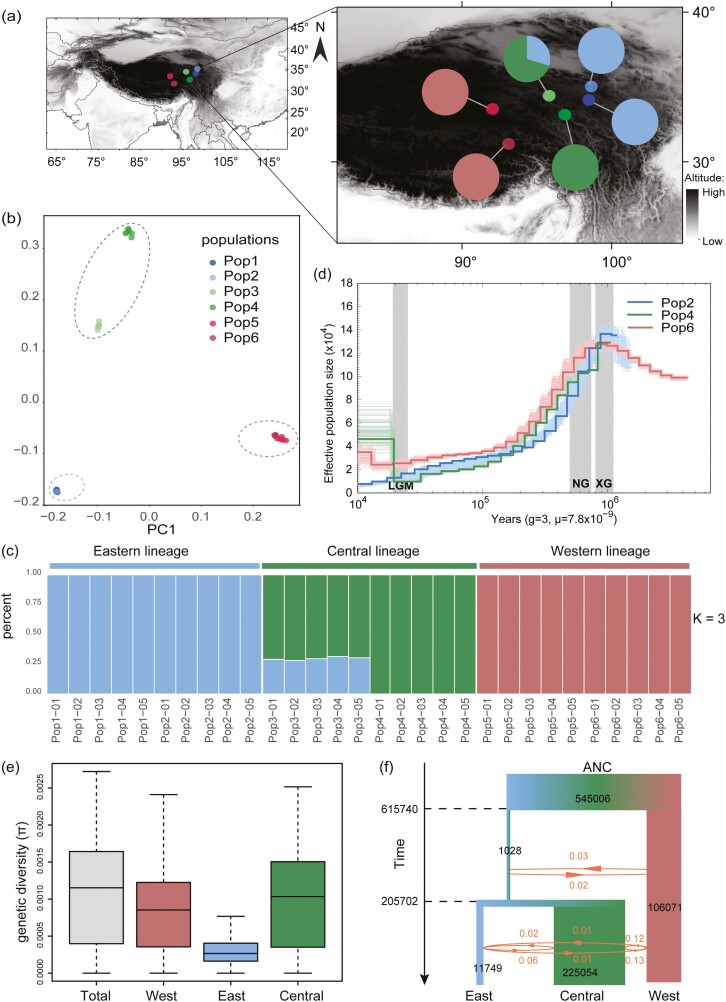
Population structure and demographic histories of *P. tangutica*. (a) Geographical distribution of the sampling locations. (b) PCA plots of the first two components. (c) Admixture analysis with individual ancestry coefficients K=3. (d) Demographic history of *P. tangutica* inferred by PSMC. The periods of the Xixiabangma Glaciation (1.17–0.8 Mya), Naynayxungla Glaciation (0.72–0.5 Mya) and the last glacial maximum (~20 thousand years ago) are shaded. (e) Genetic diversity of three lineages and the total species. (f) Demographic history simulated by fastisimcal2. Arrows and associated figures indicate direction and gene flow (per generation migration rates × *Ne*).

We performed whole-genome resequencing for 30 individuals from six populations in the core QTP (Fig. 5a). For each individual, we generated an average of 23.15-fold coverage depth, based on the reference genome (Supplementary Table S24). In total, 64,148,143 high-quality SNPs were detected and used for population structure analysis. Pairwise genetic distances of all individuals were visualized by means of a neighbour-joining tree, which revealed three distinct lineages (Supplementary Fig. S18). PCA results yielded three similar clusterings (Fig. 5b). An analysis of the population structure revealed three distinct lineages (*K* = 3) with corresponding geographical distributions (eastern, western, and central lineages) (Fig. 5c). One population (Fig. 5a) was found to arise through hybridization between western and central lineages because it contained genetic compositions of both lineages. This hybrid origin was also supported by our statistical analyses (Supplementary Table S25). The central lineage had a higher genetic diversity than the other two, while the total genetic diversity of this endangered species (Supplementary Table S26) was estimated to be smaller than those of other alpine plants occurring there.^6,89^ In addition, we estimated the inbreeding coefficient (*F*_IS_) for each sampled individual across all lineages, and it varied from 0.0005 to 0.84 between different individuals. Most *F*_IS_ values of the sampled individuals in the eastern lineage were higher than 0.7 (Supplementary Table S24). In addition, the selfing rate (*s*) values similarly varied from 0.001 to 0.91 (Supplementary Table S24). All these results suggest that self-pollination varied greatly between different populations and lineages.

To investigate the demographic history of *P. tangutica*, we performed PSMC analysis by randomly selecting one individual from each of the three lineages. The common ancestor of the three lineages had high effective population sizes (*N*_*e*_) ~950–800 thousand years ago (kya) and then followed with a sharp decline, which coincided with the development of Naynayxungla glaciation (720–500 kya)^1^ (Fig. 5d). With decreasing populations, three lineages diverged. Following the end of the Last Glacial Maximum at approximately 20 kya, the *N*_*e*_s of both the western and eastern lineages continued to decline, whereas that of the central lineage started to recover and expand. The genetic diversity (*π*) of each lineage ranged from 1.31 × 10^−^4 to 1.08 × 10^−^3 (Fig. 5e), and that of the total species was 1.22 × 10^−^3. The high genetic diversity of the central lineage is consistent with its postglacial population expansion. Three lineages were further estimated to diverge from another between 800 and 200 kya, consistent with the divergences inferred from PSMC. Gene flow between the three lineages was continuous but weak, especially between eastern and western lineages (Fig. 5f). Gene flow from both the eastern and western lineages to the central lineage was clearly higher than that in the other directions.

## 4. Discussion

In this study, we reported the high-quality chromosome-level genome sequence of one alpine plant, *P. tangutica*. We identified one lineage-specific WGD, which may have promoted the adaptation of this species to arid habitats. WGD helps plants colonize new niches and further buffers against the inbreeding effects when there are an insufficient number of pollinators.^90^ On the high-altitude QTP, alpine plants with recent WGD frequently occur, although some show no obvious increased chromosome number.^1,2^ WGD doubles the original gene sets and selectively retains those related to niche adaptation.^91^ Our genomic analyses of the QTP plant *P. tangutica* confirmed this hypothesis that many genes involved in the abiotic stress response were retained after WGD. The expansion of such genes is easier through WGD than by other ways.^92^

Because of the lack of enough pollinators, many alpine plants shift to facultative self-pollination from the closely related completely outcrossing species in the low-altitude regions.^93^ Recent genomic comparisons have revealed the underlying genetic mutations for such one alpine self-compatible plant in Brassiaceae.^5^ In this species, two key mutations that result in self-compatibility were found to be at locations of conserved structural and functional integrity of the self-incompatibility proteins. In most obligate outcrossing Solanaceae species, GSI is controlled by the S-locus that comprises two linked S-RNase and SLF genes.^55^ The GSI locus seemed to have been broken by the inserted LTR in the two S-RNase genes and the loss of the linked SLF genes in *P. tangutica* (Fig. 3). It remains unknown which occurred first. In addition, two putative RNase-like genes show no flower-specific expressions. We speculate that these changes may be related to the disruption of GSI, self-compatibility, and facultative breeding system of *P. tangutica*. This was further confirmed by the high inbreeding coefficient and selfing rate in some populations (Supplementary Table S24).

Despite the unique alpine adaptation developed for this alpine species, it has become endangered in the recent past. Our further population genomic analyses of this species reveal that this alpine plant had decreased its effective population sizes since 720–500 kya when the largest glaciation of the Quaternary occurred in the QTP.^93^ At this stage, intraspecific divergences occurred in many species likely because of distributional retreats into different refugia.^94^ Three lineages of *P. tangutica* may have also evolved because of the geographic isolation caused by the Quaternary glaciations. We found that gene flow between three lineages was continuous but very weak (Fig. 5f). In addition, genetic diversity of each lineage and of the whole species is relatively low (Fig. 5e). High inbreeding coefficients and selfing rates were also found in some populations (Supplementary Table S24). These genetic indices are largely consistent with the dominant self-pollination observed for some populations of this species in the field.^10^ However, we recovered one hybrid population between central and eastern lineages, suggesting that outcrossing may still have frequently occurred within this species. Therefore, this facultative breeding system may benefit this species in terms of both reproductive assurance from self-pollination and gene flow from outcrossing to avoid inbreeding depression.^95^ This flexible pollination mechanism is highly advantageous in arid QTP environments when effective pollinators are scarce or unstable.^96^ The continuously decreased population size and endangered status of *P. tangutica* may have resulted mainly from climatic changes since the Quaternary, although anthropogenic activities in the recent past have enforced this endangered situation.

## Supplementary Material

dsad005_suppl_Supplementary_Material

## Data Availability

All genomic sequencing data and transcriptomic raw data used in this study have been deposited in the NCBI Sequence Read Archive under BioProject accession numbers PRJNA791792 (reviewer link: https://dataview.ncbi.nlm.nih.gov/object/PRJNA791792?reviewer=4317isgri9bqv14lc4i4tjd1aj) for *Przewalskia tangutica*. Accession numbers for transcriptome data are SRR22371366 to SRR22371384. The genome assembly and annotations are available at Figshare with https://figshare.com/s/9c30e64de8610e4ed8ab.
