# Supplementary material for: The genome sequence and demographic history of Przewalskia tangutica (Solanaceae), an endangered alpine plant on the Qinghai–Tibet Plateau
Source: DNA Res. 2023 Apr 4;30(2):dsad005. doi: 10.1093/dnares/dsad005 (PMC10119639; doi:10.1093/dnares/dsad005)
Supplement: dsad005_suppl_Supplementary_Material [file dsad005_suppl_supplementary_material.docx]

**Supplementary Information**

The genome sequence and demographic history of *Przewalskia tangutica* (Solanaceae), an endangered alpine plant on the Qinghai-Tibet Plateau

# Supplementary Figures


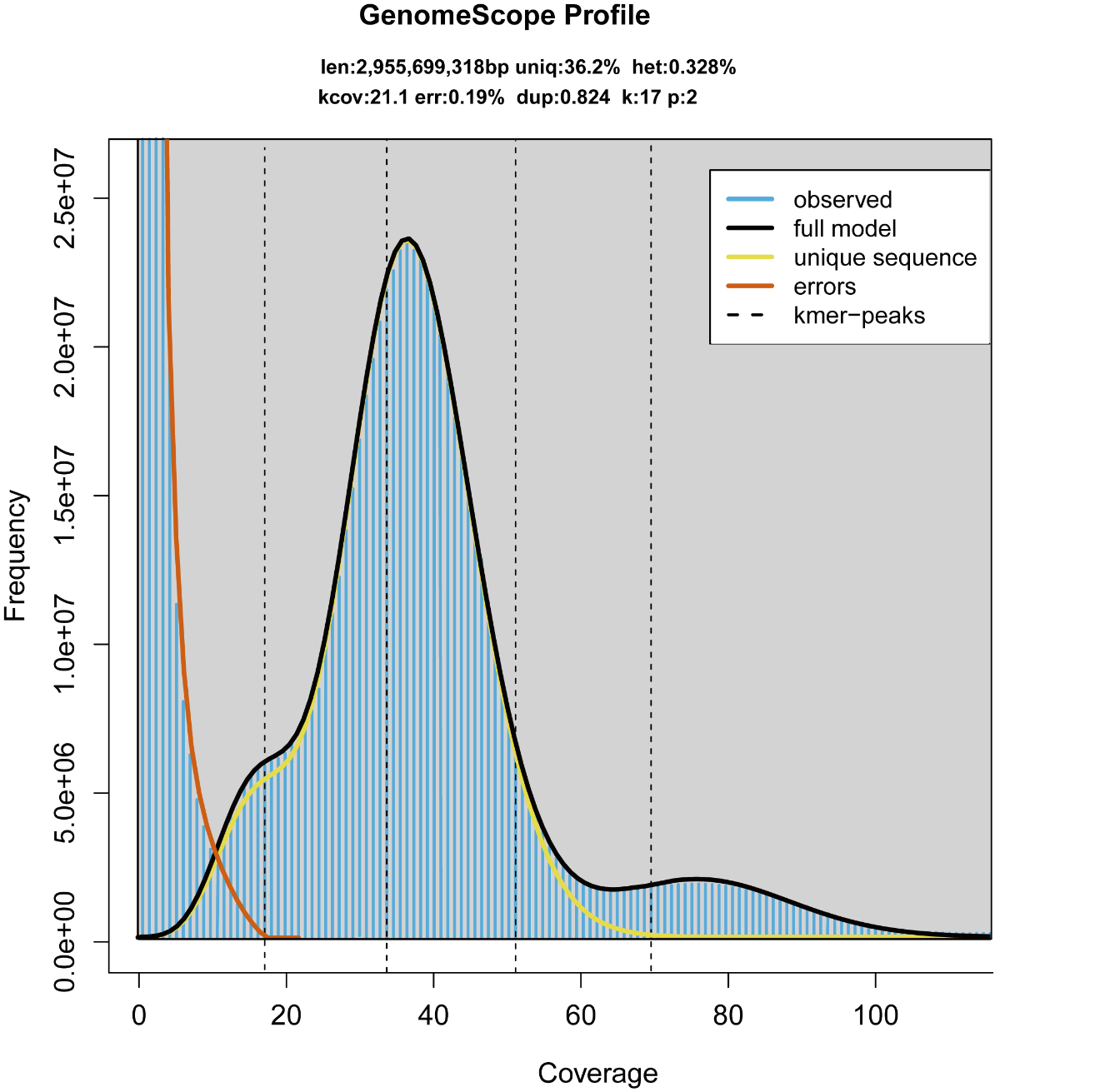


## Supplementary Figure 1. Genome size estimation by GenomeScope. *K*-mer size was set as 17 and the default parameters were used within GenomeScope

**
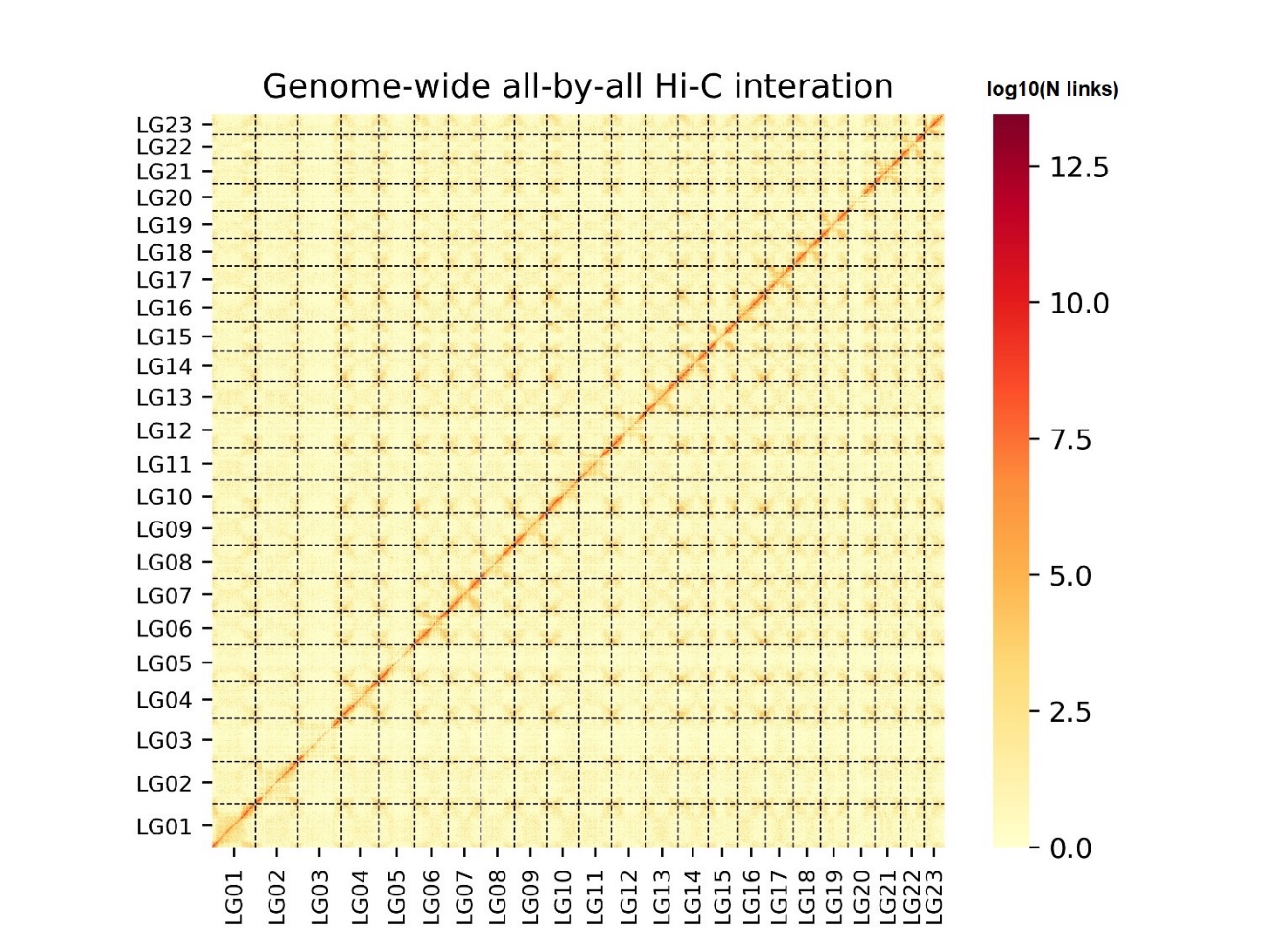
**

## **Supplementary Figure 2.** Hi-C assisted assembly of *P. tangutica* pseudochromosomes. Heatmap showing Hi-C interactions under a resolution of 200 kb, and the antidiagonal pattern for the intrachromosomal interactions may reflect the Rabl configuration of chromatins.

**
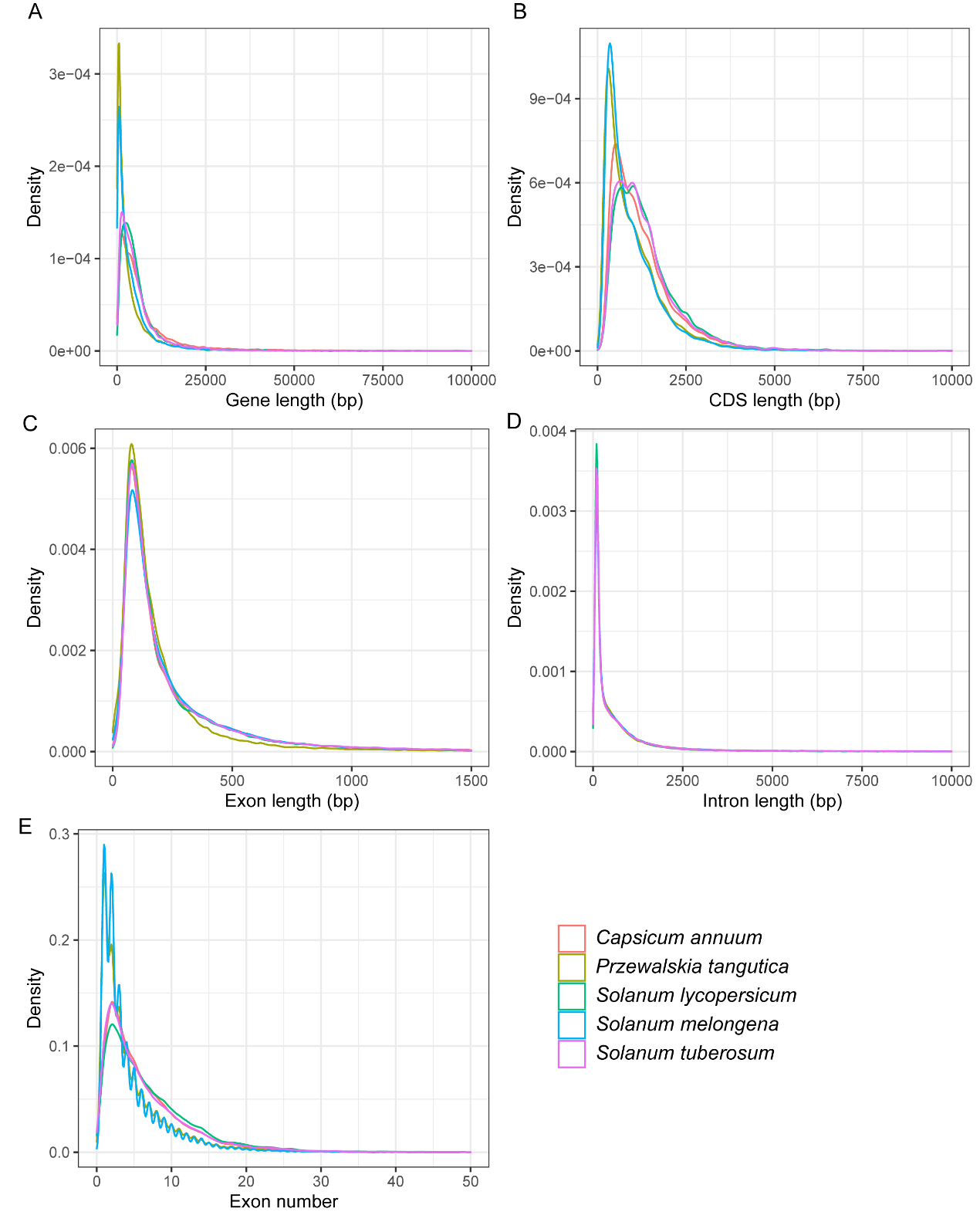
**

## **Supplementary Figure 3.** **Comparison of gene structure characteristics in *P. tangutica*, *C. annuum, S. lycopersicum, S. melongena* and *S. tuberosum*.** A, gene length. B, CDS length. C, exon length. D, intron length. E, exon number.


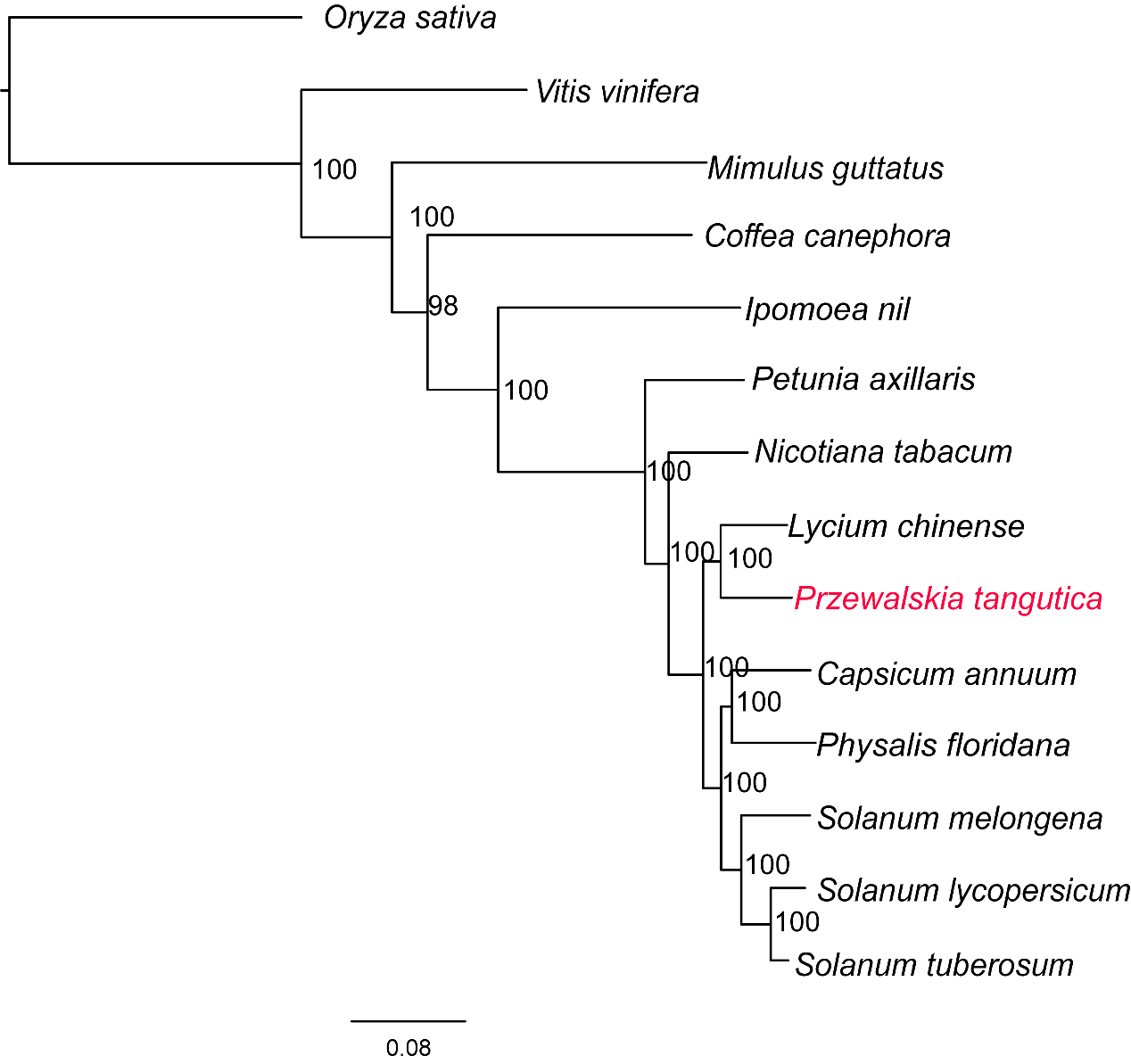


## Supplementary Figure 4. The phylogenetic tree shows the topology and bootstrap values for *P. tangutica* and other 13 plant species. Branch length represents the rate of evolution. The numbers beside the branch indicated the bootstrap value (1000 times). MUSCLE was used to generate multiple sequence alignment for protein sequences in 500 single-copy families with default parameters. All the alignment results were combined to create a super alignment matrix. RAxML with GTRGAMMA model was used to construct phylogenetic tree with maximum-likelihood algorithms. *Oryza sativa* was designated as an outgroup of the phylogenetic tree.


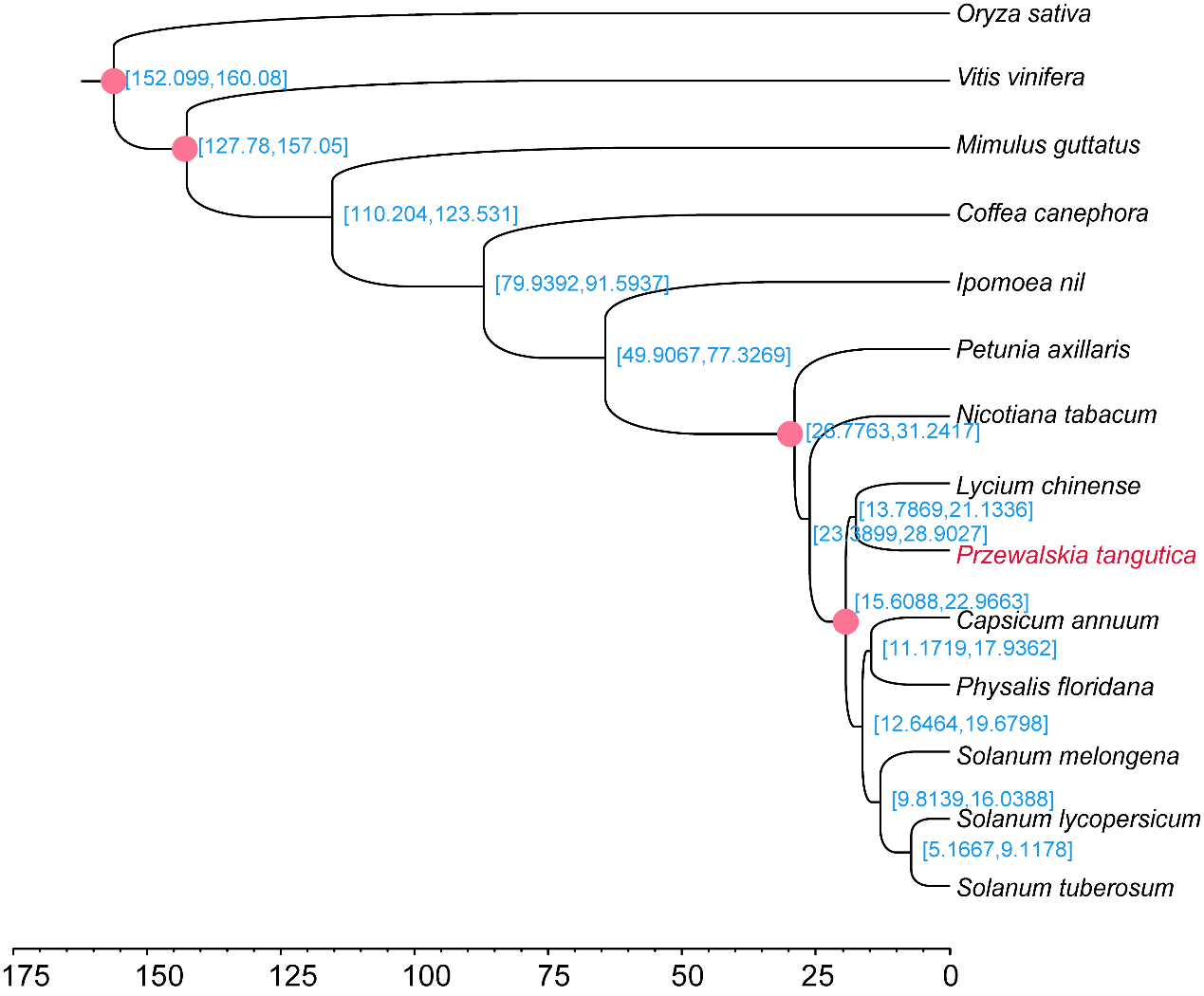


## **Supplementary Figure 5. Phylogenetic tree and divergence time estimation of *P. tangutica* and other 13 plant species.** Divergence time was estimated using the mcmctree (http://abacus.gene.ucl.ac.uk/software/paml.html) embedded in the PAML package. The most likely divergence times from the most recent comment ancestor are given along each node, and the estimated ranges of divergence times are shown in the parentheses. Numbers in the parentheses are the predicted divergence times (95% confident intervals).

**
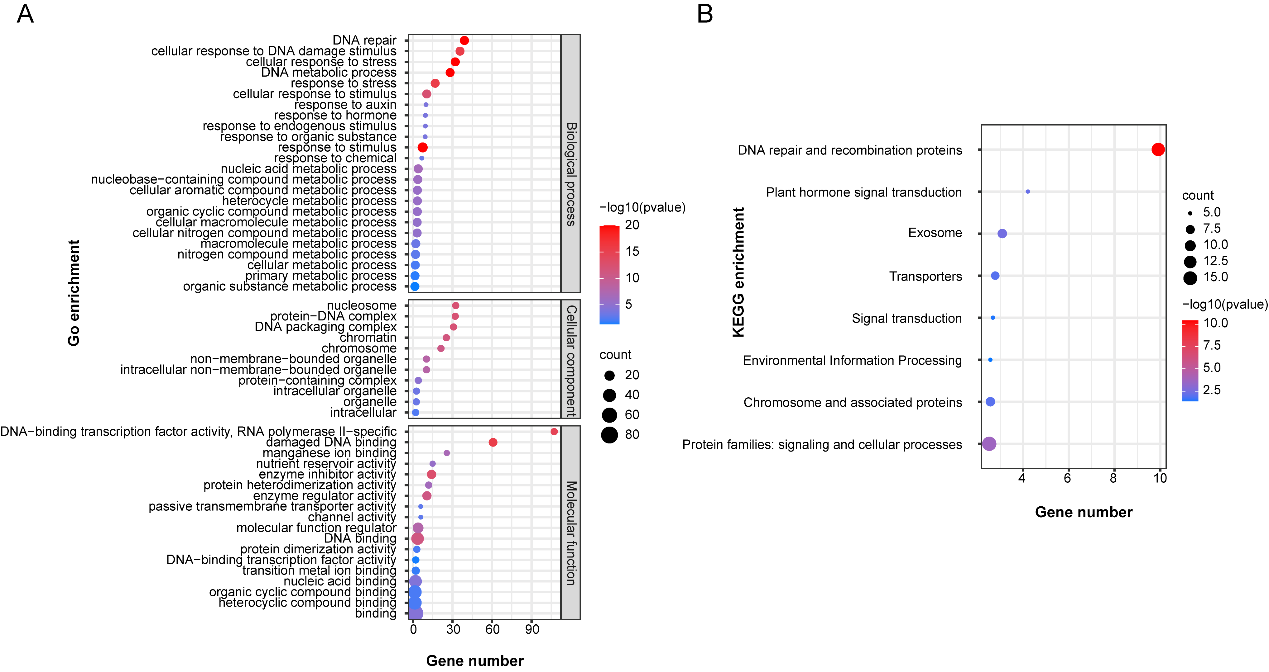
**

## Supplementary Figure 6. Enrichment analysis of significantly expanded gene families in *P. tangutica*. A, GO enrichment analyses. *P* values represent the significance of the enrichment. The source data are listed in Supplementary Table 13. B, KEGG analysis. *P* values represent the significance of the enrichment. The source data are listed in Supplementary Table 14. Circles indicate the target genes, and the size is proportional to the number of genes.


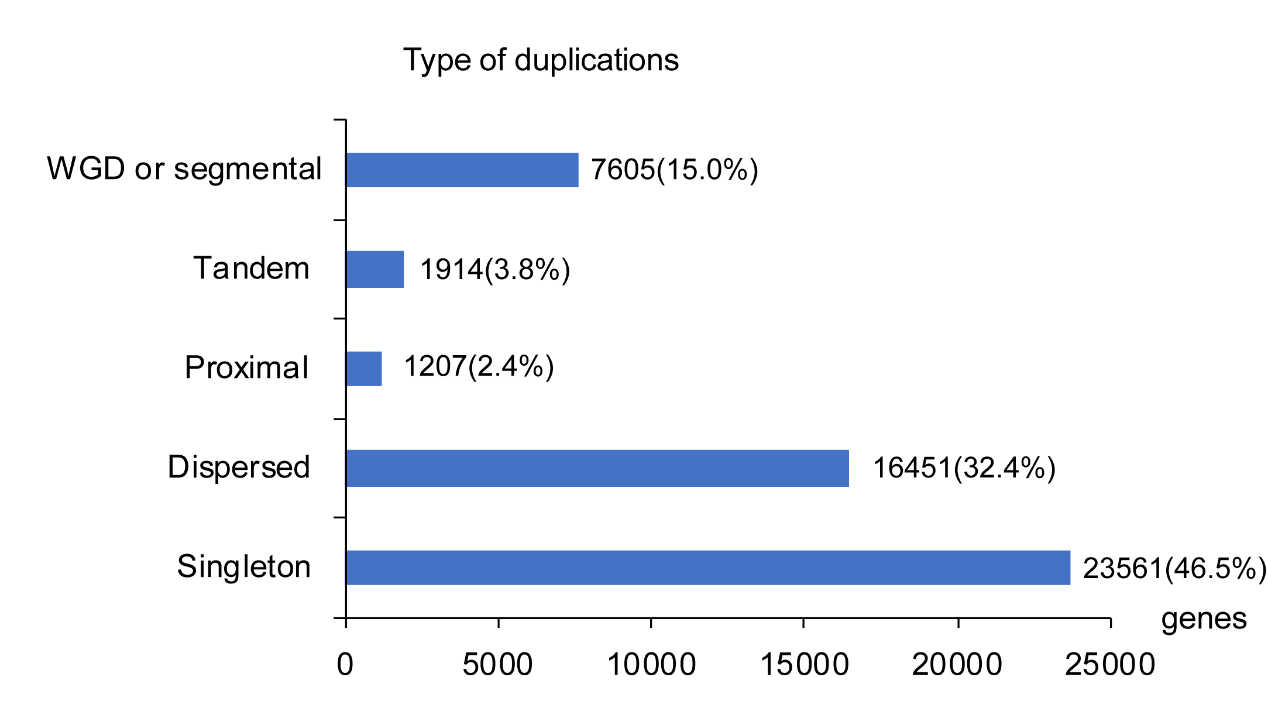


## Supplementary Figure 7. Types of gene duplication in the *P. tangutica* genome. Distribution of the five duplication types classified by MCScanX as follows: Singleton: no duplication; WGD/segmental: whole genome or segmental duplications (collinear genes in collinear blocks); Tandem: consecutive duplication; Proximal: duplications in nearby chromosomal region but not adjacent; Dispersed: duplications of modes other than tandem, proximal or WGD/segmental.


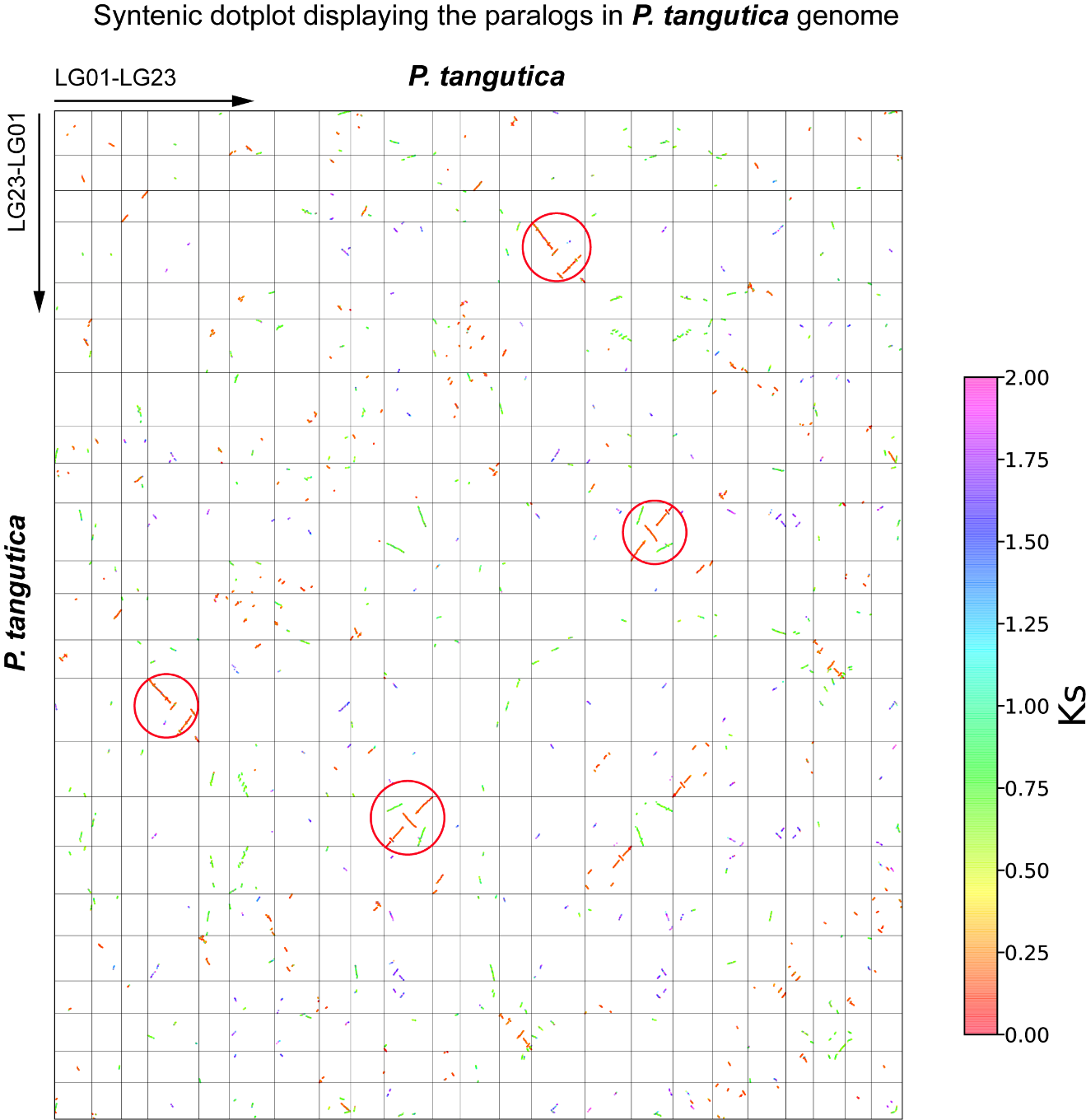


## Supplementary Figure 8. Syntenic dotplot and Ks dotplot displaying the paralogs in *P. tangutica* genome. Syntenic blocks were identified and colored based on their Ks values. Representative paralogous regions were marked out by red circle.


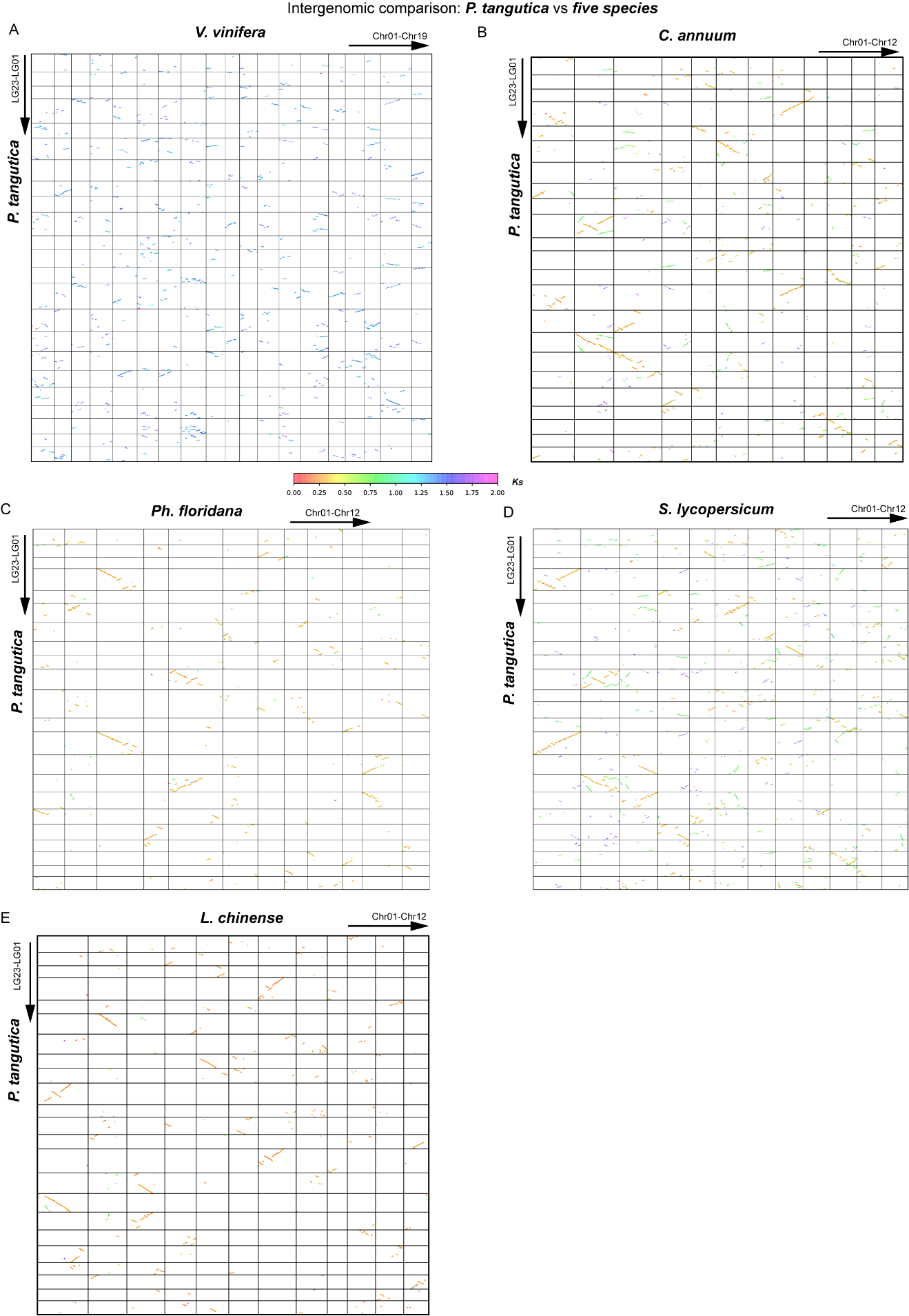


## Supplementary Figure 9. Inter-genomic syntenic block dotplot between *P. tangutica*, *V. vinifera,* *C. annuum*, *Ph. floridana*, *S. lycopersicum* and *L. chinense*. A, The syntenic block dotplot with *P. tangutica* and *V. vinifera.*B, The syntenic block dotplot with *P. tangutica* and *C. annuum.* C, The syntenic block dotplot with *P. tangutica* and *Ph. floridana.* D, The syntenic block dotplot with *P. tangutica* and *S. lycopersicum.* E, The syntenic block dotplot with *P. tangutica* and *L. chinense.*


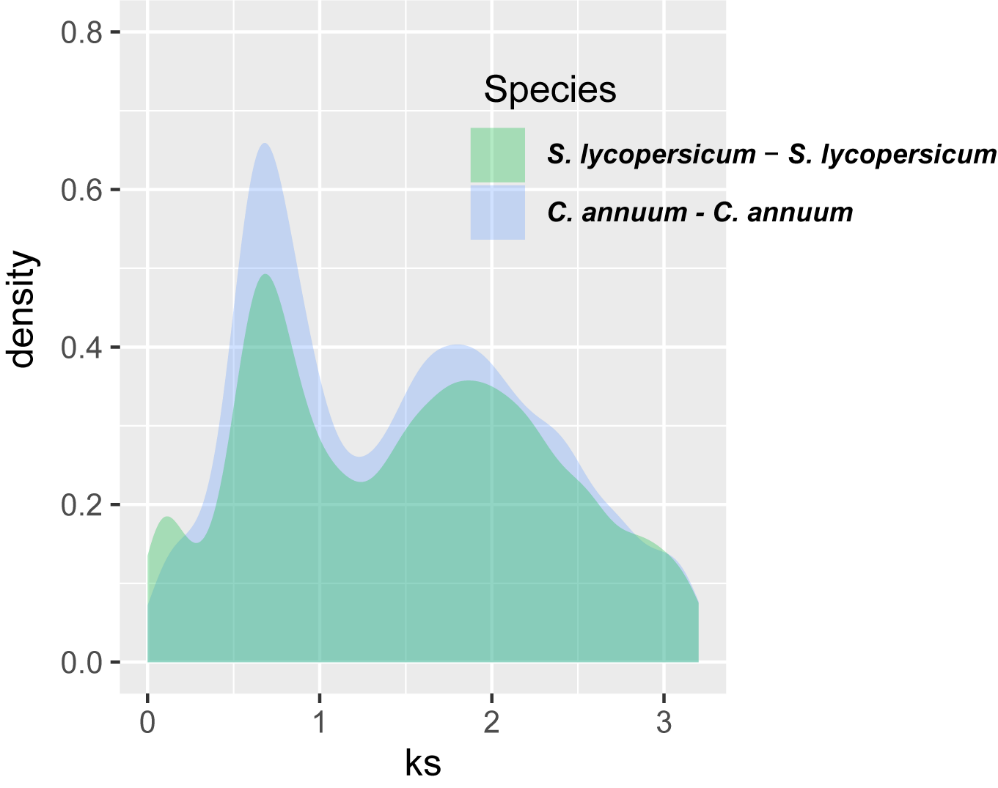


## Supplementary Figure 10. The synonymous substitution rates (Ks) distributions of paralogous genes in *C. annuum* and *S. lycopersicum*.


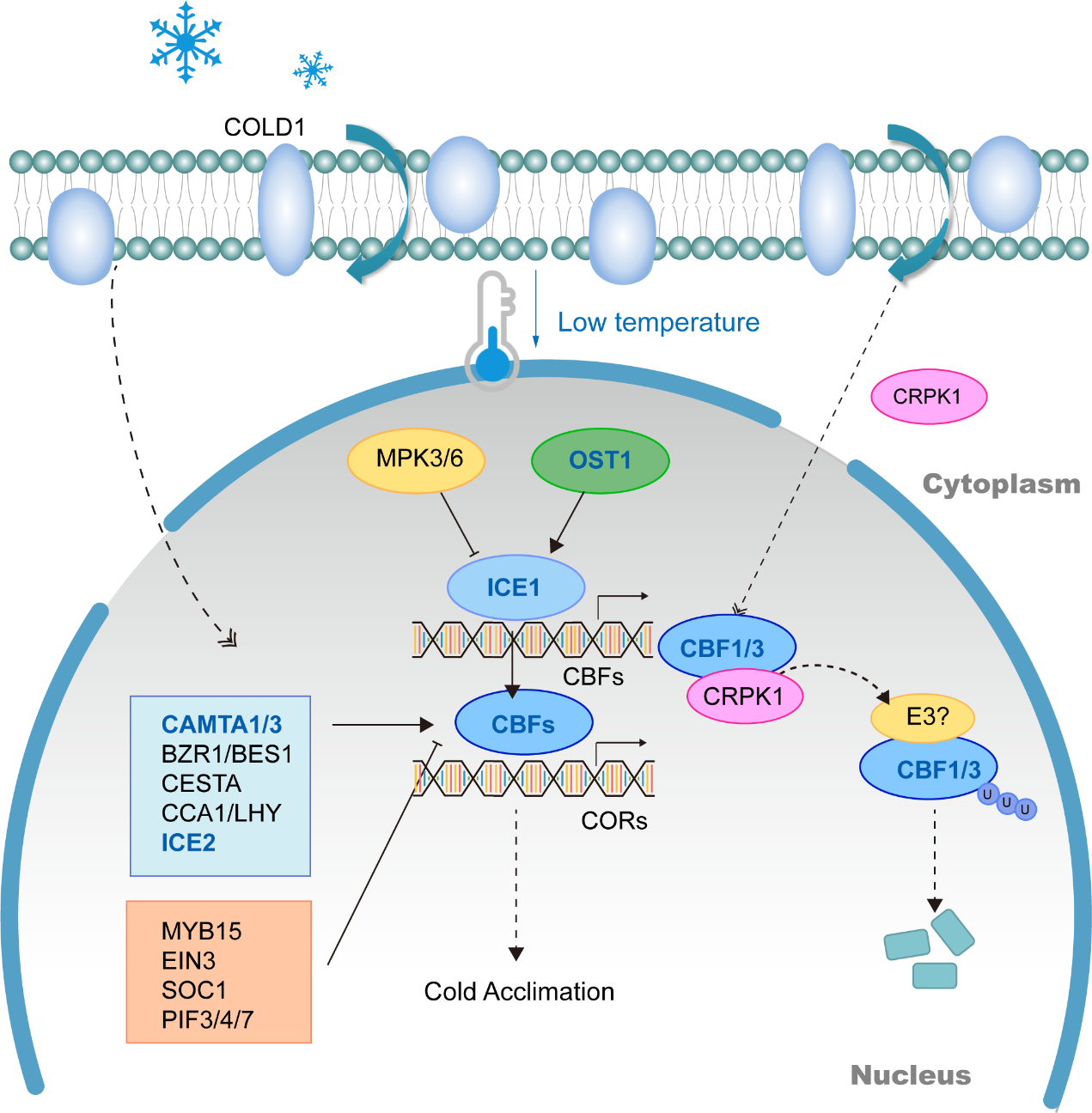


## Supplementary Figure 11. Schematic pathways in response to cold exposure in plants. Blue fonts represent genes that play a key role in the pathway.


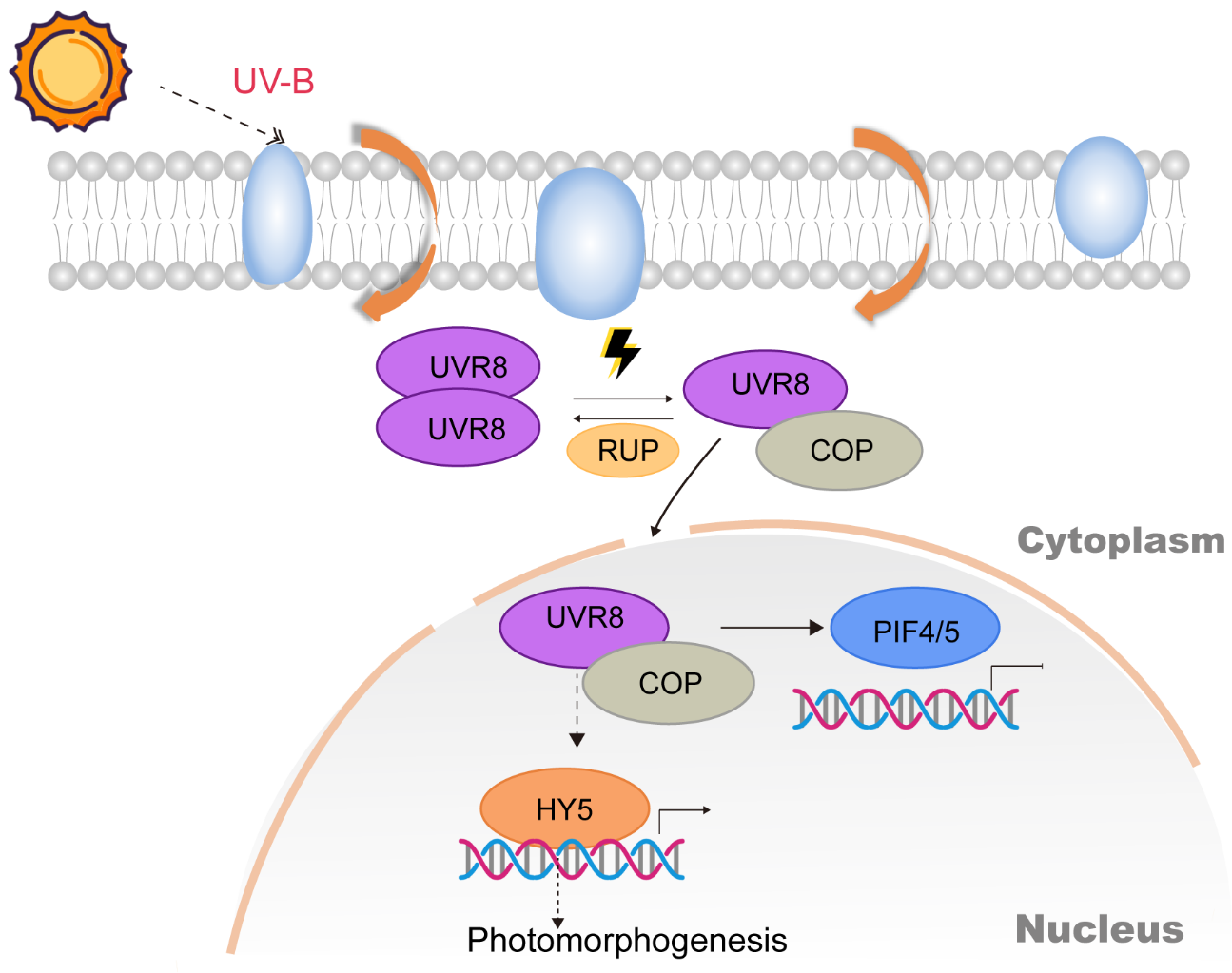


## Supplementary Figure 12. Schematic pathways in response to UV-B exposure in plants. UVR8, UV RESISTANCE LOCUS 8; COP1, CONSTITUTIVELY PHOTOMORPHOGENIC 1; HY5, ELONGATED HYPOCOTYL 5; RUP1/2, REPRESSOR OF UV-B PHOTOMORPHOGENESIS 1/2: RUP1/2 are negative regulators of the signaling pathway.


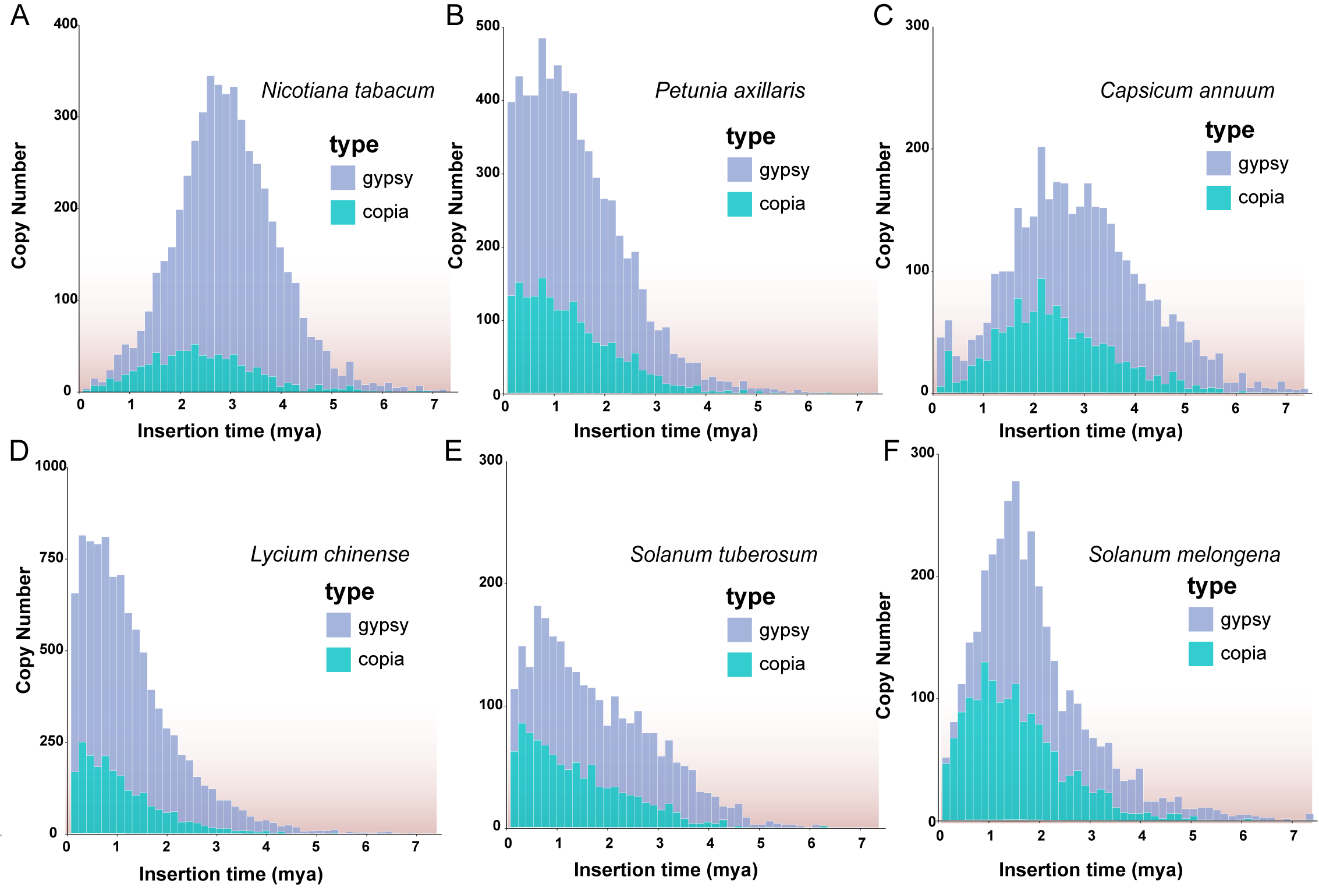


## **Supplementary Figure 13.** Distribution of insertion times of *Copia* and *Gypsy* elements in the Solanaceae species.


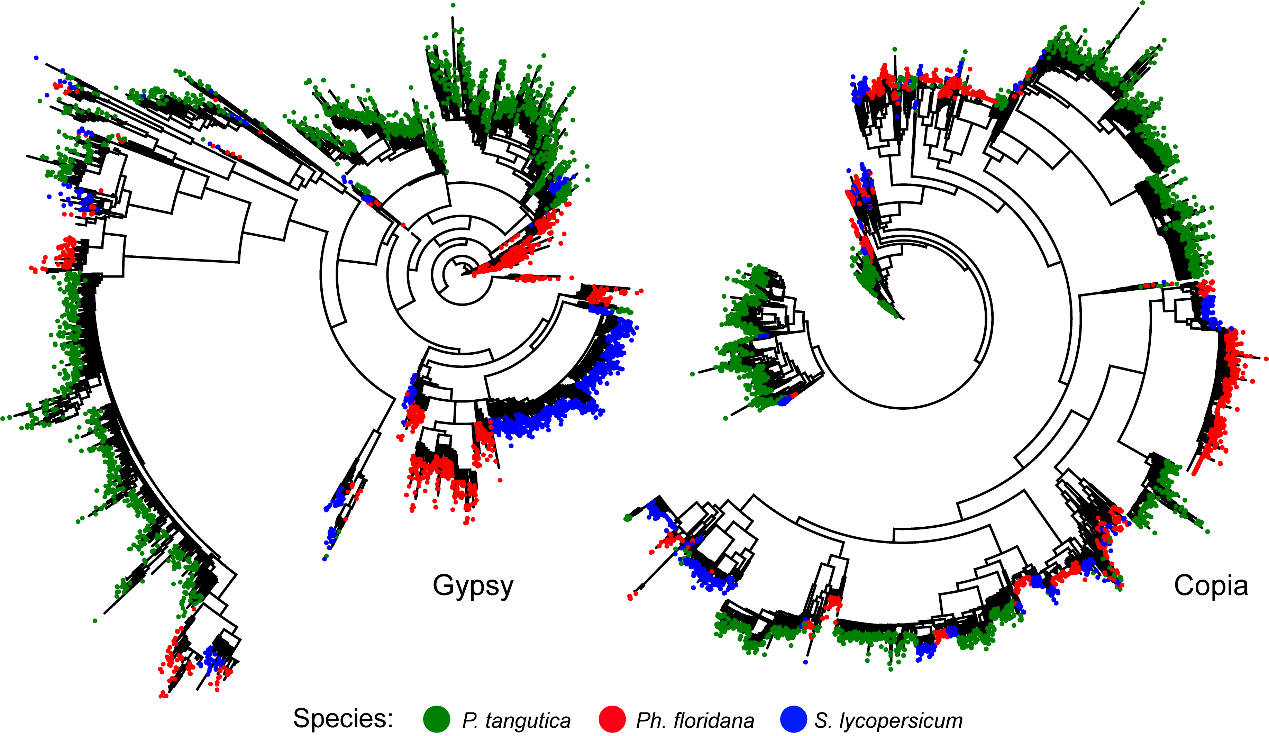


## **Supplementary Figure 14.** The unrooted phylogenetic tree of *Gypsy* and *Copia* elements was constructed on the basis of the reverse-transcriptase domain sequences.


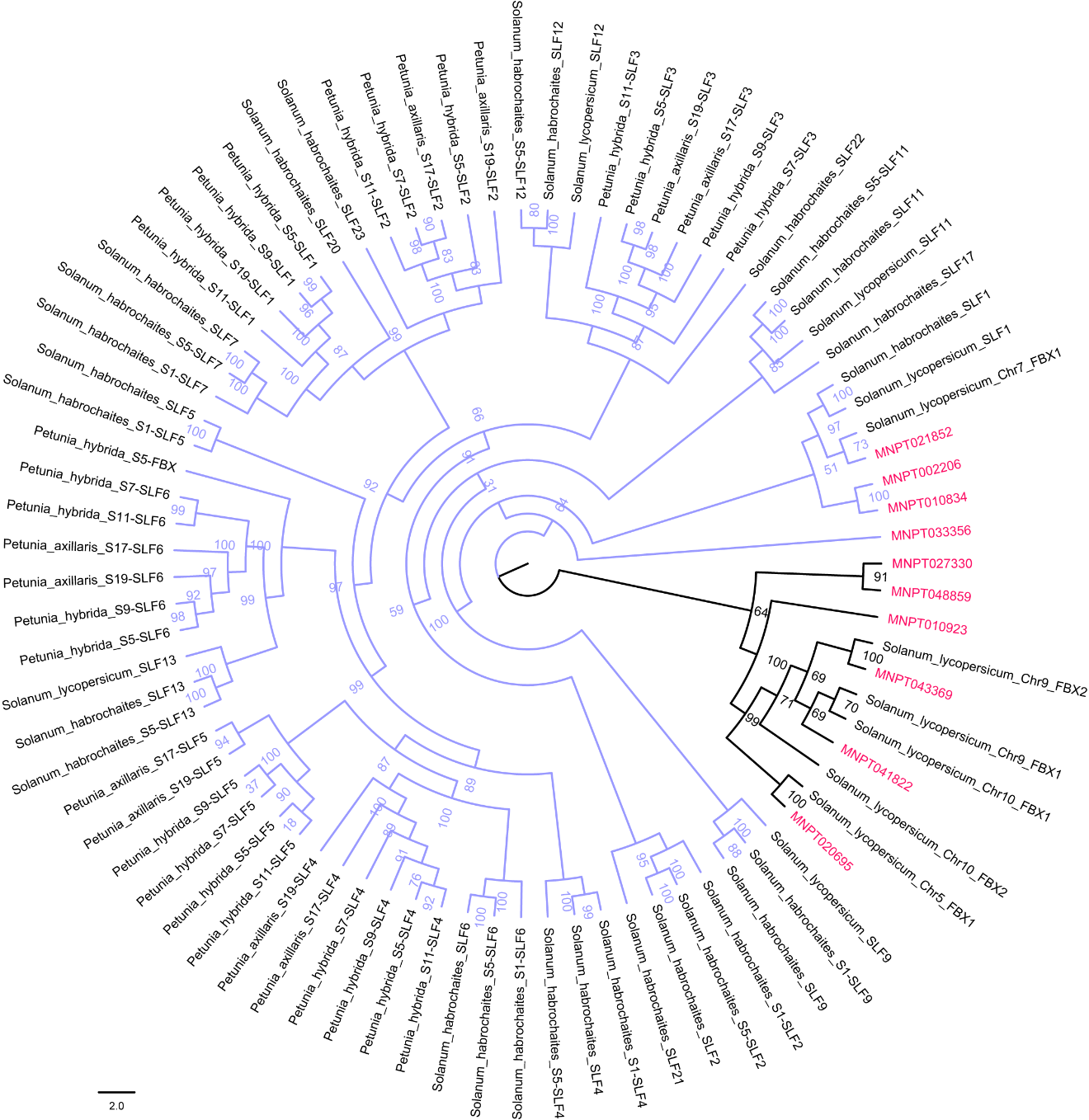


## Supplementary Figure 15. Maximum-likelihood phylogenetic tree of SLF genes of *P. tangutica* and reported SLF genes from diverse species.


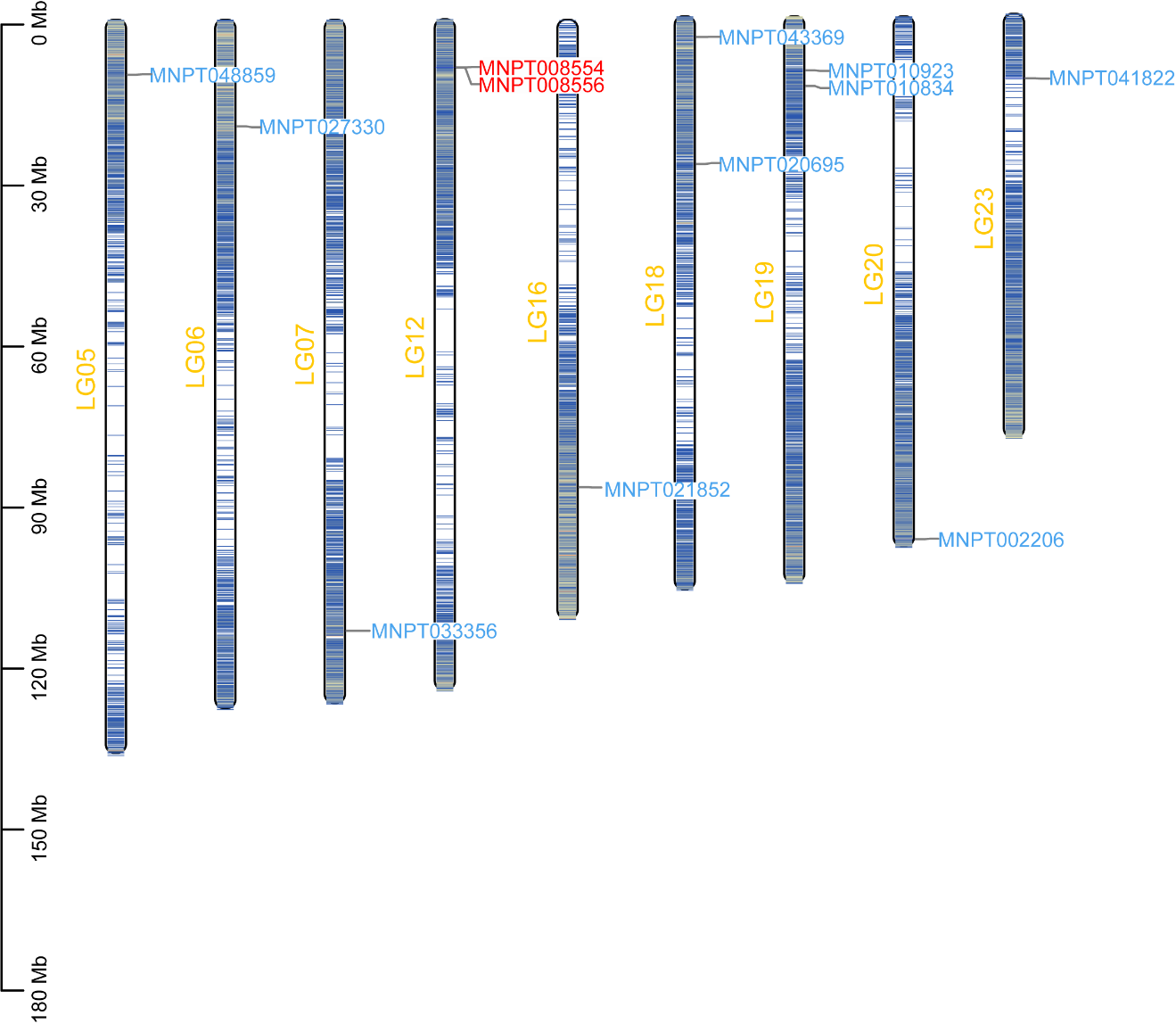


## Supplementary Figure 16. SI genes distribution along each chromosome of *P. tangutica* genome. Red color, S-RNase candidate genes; blue color, SLF genes.


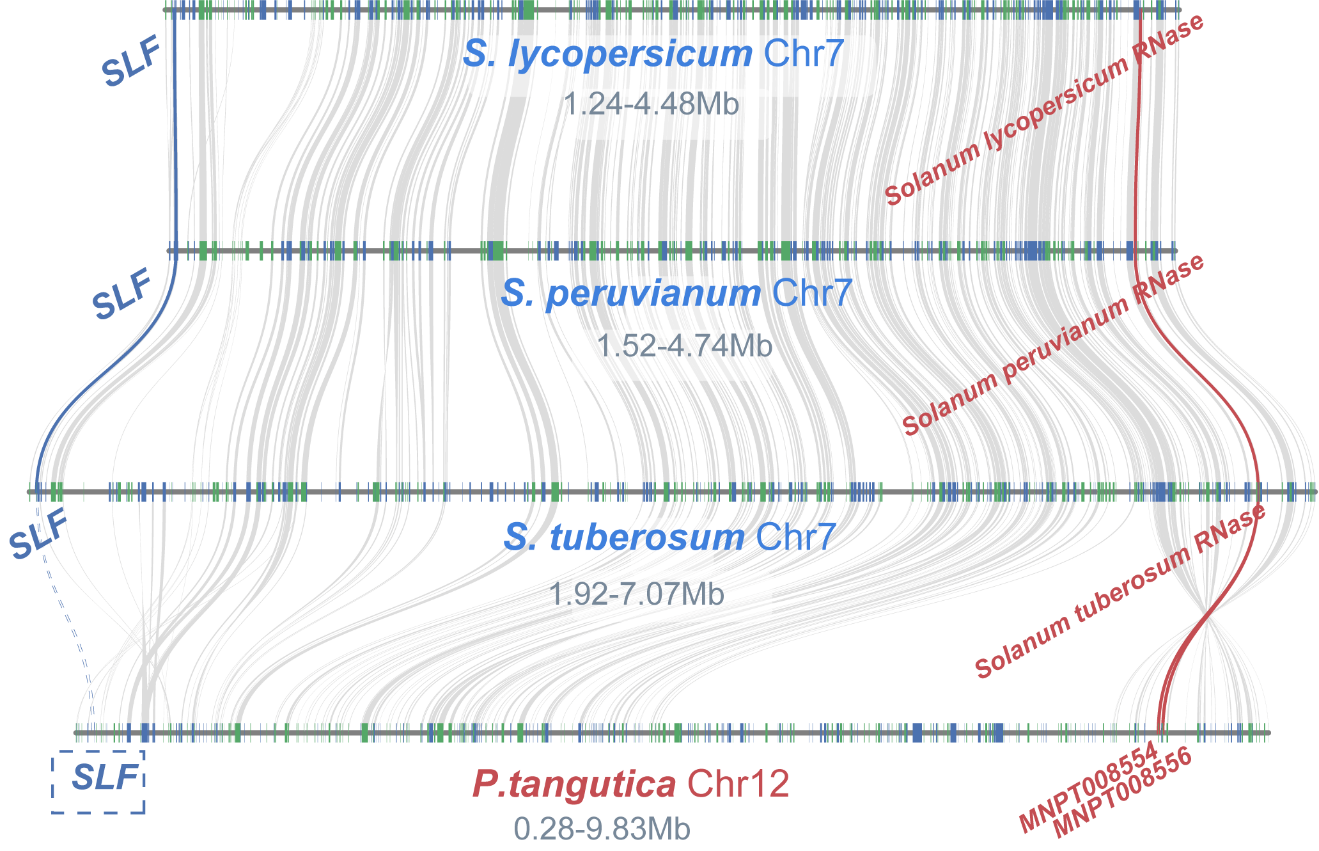


## Supplementary Figure 17. The collinearity analysis of S-locus regions in 4 chromosome level Solanaceae genomes. The lines link the syntenic S-RNase genes are highlighted in red and the SLF genes are highlighted in blue. The grey lines represent the gene collinearity among candidate species.


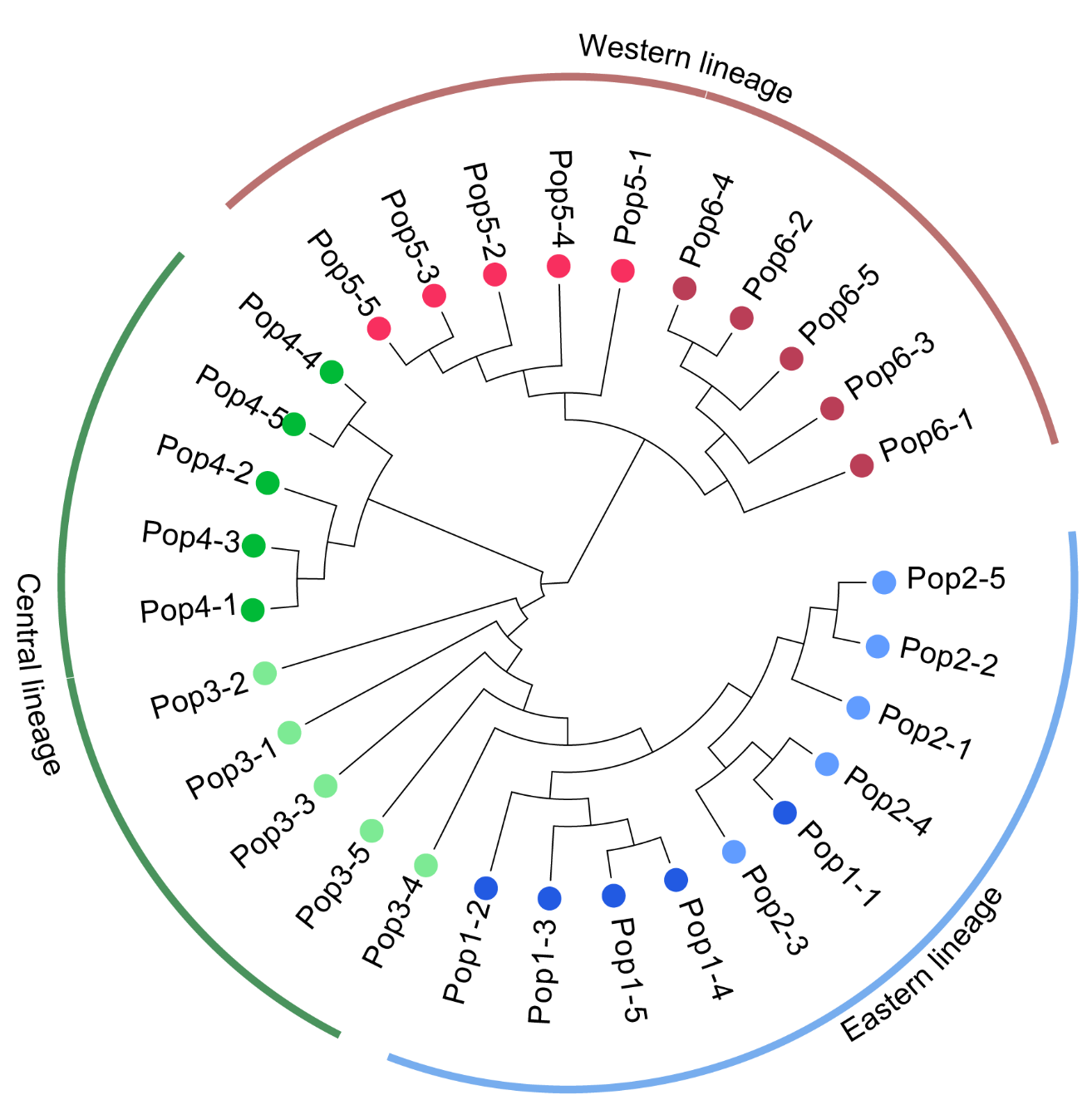


## Supplementary Figure 18. Neighbour-joining phylogenetic tree constructed using whole-genome SNP data. Different colored circles represent the 6 populations.

# Supplementary Tables

## Supplementary Table 1. The total sequencing data for *P. tangutica*

| **Type** | **Platform** | **Library type** | **Reads number** | **Data size (Gb)** | **Mean read length (bp)** | **Read N50 (bp)** | **Number** |
| --- | --- | --- | --- | --- | --- | --- | --- |
| Illumina short reads | HiSeq X (raw) | Paired | 1,392,704,454 | 159.80 | 150 | - | SRR22669370 |
|  | HiSeq X (clean)^1^ | Paired | 1,055,450,168 | 145.26 | 150 | - |  |
| ONT long reads | PromethION (raw)^1^ | Single | 17,096,605 | 271.21 | 24,126 | 34,303 | SRR22371382 |
|  | PromethION (clean) | Single | 11,493,094 | 244.47 | 21,270 | 30,280 |  |
| Hi-C reads | HiSeq X (clean) | Paired | 2,224,859,576 | 325.46 | 150 | - | SRR22350445 |

^1^The clean data means the raw data after quality control. For HiSeq reads we used fastp software with those commands: -q 20 -5 -3; for long reads we used NextDenovo to perform the error correction and obtain the final consensus sequences with the default parameters.

**Supplementary Table 2. Summary of *P. tangutica* contig leveled assembly.**

|  | **Size (bp)** | **Number** |
| --- | --- | --- |
| N90 | 733,791 | 282 |
| N50 | 17,500,920 | 49 |
| Longest | 73,151,129 | - |
| Total Contigs | 3,028,353,625 | 1610 |
| Short reads mapping rate | 99.93% | |
| Short reads mapping coverage | 74.70% | |

## Supplementary Table 3. Summary of *P. tangutica* chromosome leveled assembly.

| **Chromosome ID** | **Chromosome Length (bp)** | **Contig Number** |
| --- | --- | --- |
| LG01 | 169,662,497 | 18 |
| LG02 | 161,034,665 | 69 |
| LG03 | 157,480,115 | 171 |
| LG04 | 140,617,545 | 71 |
| LG05 | 137,212,103 | 77 |
| LG06 | 128,737,633 | 37 |
| LG07 | 127,747,210 | 12 |
| LG08 | 127,685,107 | 65 |
| LG09 | 127,084,164 | 11 |
| LG10 | 126,171,441 | 15 |
| LG11 | 125,821,780 | 38 |
| LG12 | 125,281,812 | 112 |
| LG13 | 125,175,560 | 21 |
| LG14 | 116,208,581 | 23 |
| LG15 | 114,272,948 | 15 |
| LG16 | 112,029,566 | 6 |
| LG17 | 109,025,050 | 7 |
| LG18 | 107,256,103 | 6 |
| LG19 | 105,771,349 | 38 |
| LG20 | 99,120,138 | 85 |
| LG21 | 98,619,579 | 24 |
| LG22 | 88,963,743 | 53 |
| LG23 | 79,206,522 | 8 |
| Total | 2,810,185,211 | 982 |

## Supplementary Table 4. Summary of BUSCO evaluation results.

| **Results of BUSCO evaluation for *P. tangutica* genome** | |  |
| --- | --- | --- |
| **Parameter** | **Number** | **Percent (%)** |
| Complete BUSCOs (C) | 1,580 | 97.89 |
| Complete and single-copy BUSCOs (S) | 1,241 | 76.89 |
| Complete and duplicated BUSCOs (D) | 339 | 21.00 |
| Fragmented BUSCOs (F) | 14 | 0.87 |
| Missing BUSCOs (M) | 20 | 1.24 |
| Total BUSCO groups searched | 1,614 | 100.00 |
| **Results of BUSCO evaluation for *P. tangutica* protein** |  |  |
| **Parameter** | **Number** | **Percent (%)** |
| Complete BUSCOs (C) | 1,496 | 92.69 |
| Complete and single-copy BUSCOs (S) | 1,138 | 70.51 |
| Complete and duplicated BUSCOs (D) | 358 | 22.18 |
| Fragmented BUSCOs (F) | 66 | 4.09 |
| Missing BUSCOs (M) | 22 | 1.36 |
| Total BUSCO groups searched | 1,614 | 100.00 |

## Supplementary Table 5. Gene data sets used for gene prediction analysis.

| **Species** | **Version** | **Data source/ GenBank accession number** |
| --- | --- | --- |
| *Solanum tuberosum* | 3.0 | NCBI: PRJNA63145 |
| *Solanum lycopersicum* | 3.0 | NCBI: PRJNA119 |
| *Capsicum annuum* | 1.1 | NCBI: PRJNA706772 |
| *Nicotiana tabacum* | 4.5 | https://solgenomics.net/ftp/genomes/Nicotiana_tabacum/edwards_et_al_2017/ |
| *Solanum pennellii* | 2.0 | NCBI: PRJNA256387 |
| *Arabidopsis thaliana* | Tair10 | https://phytozome.jgi.doe.gov |

| Gene set |  | Total genes predicted | Average genes length (bp) | Average CDS length (bp) | Average exon number per_gene | Average exon length (bp) | Average intron length (bp) |
| --- | --- | --- | --- | --- | --- | --- | --- |
| De novo | Augustus | 55,882 | 3,876.26 | 951.32 | 4.62 | 205.95 | 428.49 |
|  | Genscan | 60,588 | 2,743.70 | 1,012.29 | 5.76 | 175.86 | 489.57 |
|  | Glimmerhmm | 52,445 | 1,808.93 | 743.25 | 3.13 | 237.34 | 499.96 |
| Homolog | *Solanum tuberosum* | 41,966 | 4,825.99 | 1,233.98 | 5.19 | 237.76 | 857.26 |
|  | *Solanum lycopersicum* | 40,962 | 4,947.96 | 1,242.62 | 5.29 | 234.76 | 863.10 |
|  | *Capsicum annuum* | 48,043 | 4,311.06 | 1,132.54 | 4.81 | 235.48 | 834.35 |
|  | *Nicotiana tabacum* | 55,712 | 3,999.99 | 1,104.12 | 4.53 | 243.93 | 821.18 |
|  | *Solanum pennellii* | 40,548 | 4,931.67 | 1242.97 | 5.28 | 235.57 | 862.57 |
|  | *Arabidopsis thaliana* | 32,735 | 5,198.96 | 1,249.77 | 5.63 | 221.91 | 852.66 |
| RNA-seq | PASA | 57,044 | 8,672.14 | 1,088.34 | 5.31 | 205.01 | 934.61 |
| Final set | EVM | 50,828 | 4,372.30 | 1,025.65 | 4.78 | 214.67 | 885.60 |

## Supplementary Table 6. Prediction of protein coding genes in *P. tangutica* genome.

## Supplementary Table 7. Comparison of gene space of the *P. tangutica* with other genomes.

| Species | Gene Predicted | Average Gene Length_(bp) | Average CDS Length (bp) | Average Exons per Gene | Average Exon Length (bp) | Average Intron Length (bp) |
| --- | --- | --- | --- | --- | --- | --- |
| *Przewalskia tangutica* | 50,828 | 4,372.30 | 1,025.65 | 4.78 | 214.67 | 885.60 |
| *Solanum lycopersicum* | 38,023 | 5,981.04 | 1,417.34 | 7.23 | 289.89 | 1,137.13 |
| *Capsicum annuum* | 45,131 | 7,842.20 | 1,275.4 | 6.71 | 295.41 | 1,769.85 |
| *Nicotiana tabacum* | 35,519 | 4,780.75 | 1,067.46 | 4.83 | 221.15 | 950.27 |
| *Solanum melongena* | 36,567 | 4,584.63 | 990.77 | 4.31 | 229.99 | 36.57 |
| *Arabidopsis thaliana* | 48,147 | 2,661.13 | 1,299.35 | 6.36 | 281.95 | 162.19 |
| *Solanum tuberosum* | 37,885 | 5,932.47 | 1,366.75 | 6.63 | 290.84 | 871.45 |
| *Physalis floridana* | 32,075 | 4,023.45 | 1,134.17 | 4.63 | 244.89 | 796.66 |

## Supplementary Table 8. Functional annotation of the predicted genes for *P. tangutica*.

|  | **Database** | **Number** | **Percent (%)** |
| --- | --- | --- | --- |
| Total |  | 45,250 | 89.91 |
|  | InterPro | 43,689 | 86.81 |
| Annotated | GO | 26,193 | 51.53 |
|  | NR | 41,547 | 81.74 |
|  | Swiss-Prot | 31,895 | 63.37 |
|  | KEGG | 17,600 | 34.97 |
| Unannotated |  | 5,078 | 10.09 |

## Supplementary Table 9. Prediction of repetitive elements in the assembled *P. tangutica* genome.

|  | **Length(bp)** | **Rate of genome (%)** |
| --- | --- | --- |
| RepeatMasker | 529,489,405 | 17.48 |
| RepeatModeler | 2,456,662,116 | 81.12 |
| RepeatProteinMasker | 417,926,684 | 13.80 |
| TRF | 152,771,249 | 5.04 |
| Total | 2,521,629,971 | 83.27 |

## Supplementary Table 10. **Statistics for non-coding RNA genes in the** *P. tangutica* **genome.**

| **Non-coding RNAs** | **Number** | **Average Length (bp)** | **Total Length (bp)** |
| --- | --- | --- | --- |
| tRNA | 3,080 | 73.636 | 226,798 |
| rRNA | 2,887 | 165.500 | 477,799 |
| snRNA | 11,780 | 108.931 | 1,293,207 |
| miRNA | 644 | 153.990 | 99,105 |

## Supplementary Table 11. Plant genomes for comparative genomics analysis

| **Species Name** | **Abbreviation** | **Family** | **Data source/ Accession number** |
| --- | --- | --- | --- |
| *Oryza sativa* | Osa | Poaceae | NCBI: PRJNA644720 |
| *Vitis vinifera* | Vvi | Vitaceae | NCBI: PRJEA18785 |
| *Coffea canephora* | Cca | Rubiaceae | NCBI: PRJEB4211 |
| *Mimulus guttatus* | Mgu | Phrymaceae | NCBI: PRJNA285087 |
| *Ipomoea nil* | Ini | Convolvulaceae | NCBI: PRJNA344313 |
| *Capsicum annuum* | Can | Solanaceae | NCBI: PRJNA706772 |
| *Petunia axillaris* | Pax | Solanaceae | NCBI: PRJNA533335 |
| *Solanum lycopersicum* | Sly | Solanaceae | NCBI: PRJNA119 |
| *Solanum tuberosum* | Stu | Solanaceae | NCBI: PRJNA63145 |
| *Solanum melongena* | Sme | Solanaceae | https://solgenomics.net/ftp/genomes/Solanum_melongena_HQ-1315/ |
| *Nicotiana tabacum* | Nta | Solanaceae | https://solgenomics.net/ftp/genomes/Nicotiana_tabacum/edwards_et_al_2017/ |
| *Physalis floridana* | Pfl | Solanaceae | NGDC: GWHANUX00000000 |
| *Lycium chinense* | Lch | Solanaceae | unpublished |

## Supplementary Table 12. Summary of gene family clustering among the 14 genomes (including 9 Solanaceae species and 5 other angiosperms).

| **Species** | **Total genes** | **Genes in families** | **Unclustered genes** | **Families** | **Unique**  **families** | **Genes per family** | **Maximum gene famliy size** |
| --- | --- | --- | --- | --- | --- | --- | --- |
| ***P. tangutica*** | 50,828 | 35,637 | 15,191 | 20,756 | 1,427 | 1.716949 | 99 |
| ***C. annuum*** | 30,124 | 24,843 | 5,281 | 19,569 | 513 | 1.269508 | 54 |
| ***C. canephora*** | 25,571 | 19,922 | 5,649 | 15,661 | 753 | 1.272077 | 40 |
| ***M. guttatus*** | 26,688 | 22,465 | 4,223 | 15,102 | 875 | 1.487551 | 84 |
| ***I. nil*** | 35,076 | 29,515 | 5,561 | 16,728 | 1,303 | 1.764407 | 64 |
| ***L. chinense*** | 54,946 | 41,953 | 12,993 | 22,978 | 2,368 | 1.82579 | 94 |
| ***N. tabacum*** | 55,685 | 46,898 | 8,787 | 21,937 | 1,152 | 2.137849 | 114 |
| ***O. sativa*** | 24,359 | 15,483 | 8,876 | 9,733 | 1,796 | 1.590774 | 34 |
| ***Pe. axillaris*** | 32,928 | 25,659 | 7,269 | 19,566 | 634 | 1.311408 | 65 |
| ***P. floridana*** | 32,075 | 26,324 | 5,751 | 19,280 | 942 | 1.365353 | 61 |
| ***S. lycopersicum*** | 25,365 | 23,364 | 2,001 | 19,821 | 135 | 1.17875 | 44 |
| ***S. melongena*** | 36,567 | 26,406 | 10,161 | 20,603 | 959 | 1.281658 | 84 |
| ***S. tuberosum*** | 28,053 | 24,799 | 3,254 | 20,409 | 218 | 1.215101 | 23 |
| ***V. vinifera*** | 25,015 | 21,848 | 3,167 | 15,317 | 656 | 1.426389 | 56 |
| **All** | 483,280 | 385,116 | 98,164 | 40,898 | - | 9.41 | - |

## Supplementary Table 13. Gene ontology (GO) enrichment analysis of the expanded gene families in *P. tangutica*.

| **Type^1^** | **GO_Name** | **GO_ID** | ***P*-value** | **# of enriched genes** |  | **# of genes in background** |
| --- | --- | --- | --- | --- | --- | --- |
| MF | damaged DNA binding | GO:0003684 | 3.33E-16 | 13 |  | 43 |
| MF | DNA-binding transcription factor activity, RNA polymerase II-specific | GO:0000981 | 2.78E-15 | 8 |  | 15 |
| MF | enzyme inhibitor activity | GO:0004857 | 1.83E-13 | 15 |  | 214 |
| MF | DNA binding | GO:0003677 | 8.35E-12 | 36 |  | 2088 |
| MF | enzyme regulator activity | GO:0030234 | 1.21E-11 | 15 |  | 286 |
| MF | MF regulator | GO:0098772 | 1.47E-08 | 23 |  | 1188 |
| MF | manganese ion binding | GO:0030145 | 1.19E-07 | 6 |  | 47 |
| MF | protein heterodimerization activity | GO:0046982 | 3.74E-07 | 8 |  | 135 |
| MF | nutrient reservoir activity | GO:0045735 | 3.14E-06 | 6 |  | 81 |
| MF | binding | GO:0005488 | 3.41E-05 | 82 |  | 12588 |
| MF | nucleic acid binding | GO:0003676 | 5.67E-05 | 37 |  | 4000 |
| MF | passive transmembrane transporter activity | GO:0022803 | 0.001651 | 5 |  | 171 |
| MF | channel activity | GO:0015267 | 0.001651 | 5 |  | 171 |
| MF | organic cyclic compound binding | GO:0097159 | 0.006486 | 45 |  | 6544 |
| MF | heterocyclic compound binding | GO:1901363 | 0.006486 | 45 |  | 6544 |
| MF | protein dimerization activity | GO:0046983 | 0.006501 | 8 |  | 555 |
| MF | transition metal ion binding | GO:0046914 | 0.016795 | 11 |  | 1065 |
| MF | DNA-binding transcription factor activity | GO:0003700 | 0.038134 | 8 |  | 770 |
| CC | nucleosome | GO:0000786 | 6.07E-13 | 8 |  | 93 |
| CC | protein-DNA complex | GO:0032993 | 6.63E-13 | 8 |  | 94 |
| CC | DNA packaging complex | GO:0044815 | 9.38E-13 | 8 |  | 98 |
| CC | chromatin | GO:0000785 | 4.67E-12 | 8 |  | 119 |
| CC | chromosome | GO:0005694 | 2.00E-11 | 8 |  | 142 |
| CC | intracellular non-membrane-bounded organelle | GO:0043232 | 7.70E-09 | 8 |  | 295 |
| CC | non-membrane-bounded organelle | GO:0043228 | 7.70E-09 | 8 |  | 295 |
| CC | protein-containing complex | GO:0032991 | 1.29E-05 | 8 |  | 741 |
| CC | intracellular organelle | GO:0043229 | 4.23E-04 | 8 |  | 1144 |
| CC | organelle | GO:0043226 | 6.42E-04 | 8 |  | 1205 |
| CC | intracellular | GO:0005622 | 0.004404 | 8 |  | 1532 |
| BP | DNA repair | GO:0006281 | 0 | 14 |  | 106 |
| BP | response to stimulus | GO:0050896 | 0 | 19 |  | 769 |
| BP | cellular response to stress | GO:0033554 | 0 | 14 |  | 129 |
| BP | DNA metabolic process | GO:0006259 | 0 | 14 |  | 147 |
| BP | response to stress | GO:0006950 | 2.22E-16 | 14 |  | 246 |
| BP | cellular response to DNA damage stimulus | GO:0006974 | 3.33E-16 | 14 |  | 116 |
| BP | cellular response to stimulus | GO:0051716 | 5.40E-13 | 14 |  | 398 |
| BP | nucleic acid metabolic process | GO:0090304 | 2.57E-07 | 14 |  | 1047 |
| BP | nucleobase-containing compound metabolic process | GO:0006139 | 8.79E-07 | 14 |  | 1151 |
| BP | cellular aromatic compound metabolic process | GO:0006725 | 2.31E-06 | 14 |  | 1241 |
| BP | heterocycle metabolic process | GO:0046483 | 2.36E-06 | 14 |  | 1243 |
| BP | organic cyclic compound metabolic process | GO:1901360 | 3.01E-06 | 14 |  | 1267 |
| BP | cellular macromolecule metabolic process | GO:0044260 | 4.83E-06 | 14 |  | 1315 |
| BP | cellular nitrogen compound metabolic process | GO:0034641 | 4.88E-06 | 14 |  | 1316 |
| BP | response to auxin | GO:0009733 | 1.09E-04 | 5 |  | 150 |
| BP | response to endogenous stimulus | GO:0009719 | 1.35E-04 | 5 |  | 157 |
| BP | response to hormone | GO:0009725 | 1.35E-04 | 5 |  | 157 |
| BP | response to organic substance | GO:0010033 | 1.48E-04 | 5 |  | 160 |
| BP | macromolecule metabolic process | GO:0043170 | 5.96E-04 | 14 |  | 1949 |
| BP | response to chemical | GO:0042221 | 6.94E-04 | 5 |  | 223 |
| BP | nitrogen compound metabolic process | GO:0006807 | 0.001729 | 14 |  | 2138 |
| BP | cellular metabolic process | GO:0044237 | 0.006416 | 14 |  | 2407 |
| BP | primary metabolic process | GO:0044238 | 0.024771 | 14 |  | 2741 |
| BP | organic substance metabolic process | GO:0071704 | 0.043965 | 14 |  | 2907 |

^1^MF: molecular function; BP: biological process; CC: cellular component.

## Supplementary Table 14. KEGG enrichment analysis of the expanded gene families in *P. tangutica*.

| **Map ID** | **Map title** | **# of enriched genes** | **# of genes in background** | **p-value** | **Adjusted p-value** |
| --- | --- | --- | --- | --- | --- |
| A09180 | DNA repair and recombination proteins | 14 | 628 | 3.78E-11 | 1.10E-09 |
| A09180 | Protein families: signaling and cellular processes | 16 | 2823 | 2.03E-04 | 0.001965 |
| A09180 | Exosome | 8 | 1151 | 0.003568 | 0.025867 |
| A09130 | Plant hormone signal transduction | 5 | 528 | 0.006293 | 0.036501 |
| A09180 | Chromosome and associated proteins | 8 | 1384 | 0.01067 | 0.051569 |
| A09180 | Transporters | 7 | 1118 | 0.011372 | 0.041222 |
| A09130 | Signal transduction | 5 | 830 | 0.037191 | 0.119837 |
| A09130 | Environmental Information Processing | 5 | 865 | 0.043216 | 0.125328 |

## Supplementary Table 15. Transcription factor families in 5 plant genomes.

| **Gene Family** | **Description** | **No. of genes** | | | | |
| --- | --- | --- | --- | --- | --- | --- |
|  |  | ***P. tangutica*** | ***C. annuum*** | ***S. lycopersicum*** | ***S. melongena*** | ***A. thaliana*** |
| AP2 | ethylene-responsive  element binding proteins | 41 | 14 | 22 | 27 | 18 |
| ARF | Auxin response factors | 31 | 14 | 20 | 22 | 22 |
| ARR-B | response regulator | 23 | 7 | 15 | 14 | 14 |
| B3 | B3 family protein | 68 | 47 | 67 | 70 | 66 |
| BBR-BPC | BASIC PENTACYSTEINE | 12 | 6 | 6 | 6 | 7 |
| BES1 | BRI1-EMS-SUPPRESSOR 1 | 13 | 7 | 9 | 9 | 8 |
| C2H2 | C2H2 zinc finger protein | 153 | 69 | 96 | 108 | 100 |
| C3H | zinc finger CCCH domain-containing protein | 74 | 39 | 47 | 46 | 49 |
| CAMTA | Calmodulin-binding transcription activator | 9 | 5 | 7 | 7 | 6 |
| CO-like | zinc finger protein CONSTANS-LIKE | 21 | 7 | 15 | 15 | 17 |
| CPP | cystein-rich polycomb-like protein | 7 | 5 | 4 | 4 | 8 |
| DBB | double B-box zinc finger protein | 13 | 6 | 7 | 8 | 8 |
| Dof | DNA binding with one finger | 57 | 28 | 35 | 39 | 36 |
| E2F/DP | E2 factor family | 15 | 6 | 9 | 5 | 8 |
| EIL | Ethylene-insensitive3 -like proteins | 13 | 4 | 8 | 9 | 6 |
| ERF | ethylene response factors | 209 | 84 | 133 | 149 | 122 |
| FAR1 | fatty acid reductase 1 | 27 | 9 | 29 | 27 | 17 |
| G2-like | GOLDEN2-like protein | 87 | 35 | 50 | 60 | 42 |
| GATA | GATA type zinc finger transcription factor family protein | 49 | 22 | 32 | 29 | 30 |
| GRAS | GRAS family transcription factor | 87 | 50 | 49 | 56 | 34 |
| GRF | GROWTH-REGULATING FACTOR | 17 | 6 | 12 | 9 | 9 |
| GeBP | GLABROUS1 enhancer-binding protein | 12 | 2 | 10 | 15 | 22 |
| HB-PHD | Homeobox gene (HB)-encoded transcription factors (plant homeodomain with a finger domain) | 4 | 2 | 2 | 2 | 2 |
| HB-other | Homeobox gene (HB)-encoded transcription factors (without characteristic domains) | 13 | 13 | 10 | 8 | 7 |
| HD-ZIP | Homeobox gene (HB)-encoded transcription factors (homeodomain leucine zipper) | 89 | 33 | 60 | 54 | 48 |
| HRT-like | Hordeum Repressor Transcription | 1 | 1 | 1 | 1 | 2 |
| HSF | Heat stress transcription factors | 24 | 25 | 27 | 28 | 20 |
| LBD | the lateral organ boundary domains (LBD) gene family | 75 | 33 | 41 | 49 | 43 |
| LFY | LEAFY transcription factor family | 3 | 1 | 1 | 1 | 1 |
| LSD | LSD zinc finger family protein | 6 | 3 | 4 | 3 | 3 |
| M-type_MADS | K-box region and MADS-box transcription factor family protein | 62 | 27 | 78 | 67 | 66 |
| MIKC_MADS | K-box region and MADS-box transcription factor family protein | 31 | 28 | 44 | 48 | 42 |
| MYB | MYB transcription factor family protein | 182 | 102 | 137 | 131 | 144 |
| MYB_relatd | MYB_relatd transcription factor family protein | 125 | 56 | 62 | 78 | 61 |
| NAC | NAM, ATAF, and CUC (NAC) transcription factors family protein | 170 | 63 | 90 | 105 | 112 |
| NF-X1 | NF-X1 transcription factor | 3 | 2 | 2 | 1 | 2 |
| NF-YA | NF-Y transcription factor (subunits NF-YA) | 18 | 8 | 10 | 12 | 10 |
| NF-YB | NF-Y transcription factor (subunits NF-YB) | 27 | 14 | 24 | 35 | 13 |
| NF-YC | NF-Y transcription factor (subunits NF-YC) | 17 | 8 | 18 | 15 | 14 |
| Nin-like | NIN-like protein | 15 | 28 | 13 | 12 | 14 |
| RAV | RELATED TO ABI3/VP1 (RAV) protein family | 6 | 2 | 3 | 3 | 6 |
| S1Fa-like | S1FA-like DNA-binding protein | 1 | 1 | 2 | 2 | 3 |
| SAP | F-box protein STERILE APETALA | 4 | 1 | 2 | 2 | 1 |
| SBP | SQUAMOSA Promoter Binding Protein family | 31 | 14 | 16 | 12 | 17 |
| SRS | SHI-related sequence family | 14 | 4 | 7 | 7 | 11 |
| STAT | signal transducer and activator of transcription | 1 | 1 | 1 | 1 | 2 |
| TALE | Three-amino-loop-extension superfamily | 33 | 16 | 22 | 21 | 21 |
| TCP | TCP transcription factor | 38 | 18 | 30 | 30 | 24 |
| Trihelix | Trihelix DNA-binding factors | 50 | 26 | 32 | 33 | 39 |
| VOZ | Vascular plant one zinc-finger proteins | 4 | 2 | 2 | 2 | 2 |
| WOX | WUSCHEL-related homeobox proteins | 14 | 12 | 11 | 10 | 16 |
| WRKY | WRKY transcription factor family | 109 | 49 | 78 | 77 | 72 |
| Whirly | Whirly proteins | 4 | 3 | 2 | 2 | 3 |
| YABBY | YABBY transcription factor family | 12 | 6 | 9 | 9 | 6 |
| ZF-HD | Zinc finger homeodomain transcription factor family | 29 | 11 | 16 | 15 | 17 |
| bHLH | Basic Helix-Loop-Helix (bHLH) Transcription Factors | 230 | 121 | 161 | 151 | 153 |
| bZIP | basic leucine zipper (bZIP) transcription factor | 125 | 45 | 68 | 69 | 74 |

Red fonts represent transcription factor families expanding in *P. tangutica* genome.

## Supplementary Table 16. Statistics of cold resistance gene famlily in six species.

| Gene family | No. of genes | | | | | |
| --- | --- | --- | --- | --- | --- | --- |
|  | *A. thaliana* | *C. annuum* | *L. chinense* | *P. tangutica* | *Ph. floridana* | *S. lycopersicum* |
| BES1 | 4 | 4 | 6 | 9 | 5 | 5 |
| BIN2 | 10 | 8 | 11 | 16 | 9 | 11 |
| BTF3 | 2 | 2 | 2 | 4 | 2 | 2 |
| BZR1 | 4 | 5 | 8 | 10 | 6 | 6 |
| CAMTA1 | 2 | 2 | 2 | 4 | 2 | 2 |
| CAMTA2 | 3 | 2 | 2 | 4 | 2 | 2 |
| CAMTA3 | 2 | 2 | 2 | 4 | 2 | 2 |
| CAMTA5 | 1 | 4 | 6 | 5 | 4 | 2 |
| CBF | 9 | 12 | 27 | 13 | 12 | 13 |
| CCA1 | 2 | 0 | 0 | 0 | 0 | 0 |
| CESTA | 3 | 3 | 1 | 4 | 3 | 1 |
| COR | 16 | 3 | 4 | 5 | 4 | 3 |
| CRPK1 | 207 | 243 | 308 | 282 | 198 | 199 |
| EBF1_2 | 2 | 4 | 6 | 6 | 5 | 5 |
| EIL1 | 6 | 8 | 6 | 8 | 8 | 8 |
| EIN3 | 6 | 8 | 5 | 9 | 8 | 8 |
| HCHIB | 4 | 9 | 13 | 10 | 6 | 10 |
| HOS1 | 1 | 1 | 1 | 2 | 1 | 1 |
| HSFA1A | 16 | 17 | 13 | 14 | 10 | 17 |
| HY5 | 2 | 1 | 1 | 1 | 0 | 1 |
| ICE1 | 4 | 5 | 7 | 7 | 6 | 6 |
| ICE2 | 4 | 5 | 7 | 6 | 6 | 6 |
| JAZ1 | 2 | 1 | 2 | 2 | 1 | 2 |
| LHY | 3 | 0 | 1 | 0 | 0 | 1 |
| LUX | 5 | 2 | 4 | 5 | 4 | 3 |
| MPK3 | 21 | 18 | 24 | 30 | 15 | 18 |
| MPK6 | 23 | 20 | 23 | 30 | 15 | 19 |
| MYB15 | 26 | 24 | 40 | 41 | 26 | 27 |
| NPR1 | 2 | 2 | 3 | 3 | 2 | 2 |
| OST1 | 37 | 31 | 44 | 50 | 33 | 33 |
| PGIP1 | 3 | 4 | 2 | 0 | 1 | 3 |
| PIF3 | 1 | 1 | 0 | 0 | 0 | 0 |
| PIF4 | 2 | 0 | 0 | 0 | 0 | 0 |
| PIF7 | 1 | 1 | 1 | 0 | 1 | 0 |
| PUB25_26 | 4 | 2 | 4 | 4 | 4 | 2 |
| RCF1 | 6 | 8 | 7 | 14 | 7 | 7 |
| RVE8 | 5 | 3 | 3 | 6 | 3 | 3 |
| SIZ1 | 1 | 2 | 3 | 4 | 0 | 3 |
| STA1 | 2 | 1 | 1 | 1 | 1 | 1 |
| SOC1 | 9 | 7 | 9 | 6 | 2 | 7 |
| PHYB | 5 | 5 | 5 | 8 | 5 | 5 |

Red fonts represent gene families expanding in *P. tangutica* genome

## Supplementary Table 17. Statistics of DNA damage repair gene famlily in six species.

| Gene family | No. of genes | | | | | |
| --- | --- | --- | --- | --- | --- | --- |
|  | *A. thaliana* | *C. annuum* | *L. chinense* | *P. tangutica* | *Ph. floridana* | *S. lycopersicum* |
| AGO2 | 2 | 1 | 1 | 5 | 1 | 3 |
| ATM | 1 | 2 | 3 | 6 | 1 | 1 |
| ATR | 1 | 1 | 1 | 1 | 2 | 1 |
| ATRIP | 1 | 0 | 0 | 1 | 0 | 0 |
| BRCA1 | 11 | 11 | 12 | 17 | 11 | 8 |
| CYCB1 | 10 | 12 | 18 | 27 | 11 | 9 |
| NAC103 | 65 | 51 | 75 | 96 | 48 | 53 |
| PARP1_2 | 2 | 2 | 6 | 2 | 16 | 3 |
| PHR1 | 1 | 1 | 2 | 2 | 1 | 1 |
| RAD17_51_54 | 4 | 6 | 12 | 9 | 4 | 4 |
| RBR1 | 1 | 1 | 1 | 3 | 1 | 1 |
| RPA1E | 3 | 2 | 2 | 3 | 2 | 2 |
| SMR4_5_7 | 5 | 0 | 3 | 2 | 2 | 0 |
| SOG1 | 7 | 5 | 5 | 9 | 4 | 4 |
| UVR3 | 1 | 1 | 1 | 1 | 1 | 1 |
| WEE1 | 1 | 2 | 4 | 2 | 2 | 3 |

Red fonts represent gene families expanding in *P. tangutica* genome

| Gene family | No. of genes | | | | | |
| --- | --- | --- | --- | --- | --- | --- |
|  | *A. thaliana* | *C. annuum* | *L. chinense* | *P. tangutica* | *Ph. floridana* | *S. lycopersicum* |
| COP1 | 9 | 8 | 9 | 11 | 10 | 8 |
| HY5 | 14 | 12 | 18 | 32 | 11 | 22 |
| RUP1 | 2 | 1 | 2 | 1 | 1 | 1 |
| RUP2 | 2 | 1 | 1 | 3 | 1 | 1 |
| UVR8 | 1 | 1 | 0 | 2 | 0 | 0 |

## Supplementary Table 18. Number of genes involved in the UV-B response pathway in six species.

Red fonts represent gene families expanding in *P. tangutica* genome.

## Supplementary Table 19. Prediction of transposable element in the assembled *P. tangutica* genomes.

| **Type/Subfamily** |  |  | **Length (bp)** | **% of repeat** | **% of genome** |
| --- | --- | --- | --- | --- | --- |
| **Class I: DNA Transposon** | DNA  elements |  | 62,865,553 | 2.495 | 2.076 |
|  |  | DNA_CMC-EnSpm | 4,533,446 | 0.18 | 0.15 |
|  |  | DNA_MuDR | 4,053,942 | 0.161 | 0.134 |
|  |  | DNA_PIF-Harbinger | 3,466,909 | 0.138 | 0.114 |
|  |  | DNA_TcMar-Stowaway | 3,962,945 | 0.157 | 0.131 |
|  |  | DNA_hAT-Ac | 37,618,131 | 1.493 | 1.242 |
|  |  | DNA_hAT-Tip100 | 2,850,616 | 0.113 | 0.094 |
|  |  | DNA_other | 7,017,977 | 0.279 | 0.232 |
| **Class II: Retrotransposon** | LINEs |  | 53,072,407 | 2.106 | 1.753 |
|  |  | LINE_L1 | 30,191,419 | 1.198 | 0.997 |
|  |  | LINE_L2 | 95,485 | 0.004 | 0.003 |
|  |  | LINE_other | 22,895,260 | 0.909 | 0.756 |
|  | LTR elements |  | 1,971,615,678 | 78.243 | 65.105 |
|  |  | LTR_Copia | 287,655,986 | 11.416 | 9.499 |
|  |  | LTR_Gypsy | 1,672,593,866 | 66.376 | 55.231 |
|  |  | LTR_other | 14,499,197 | 0.575 | 0.479 |
|  |  | Low_complexity | 2,121,209 | 0.084 | 0.07 |
|  | SINEs |  | 3,168,965 | 0.126 | 0.105 |
|  |  | Satellite | 417,720 | 0.017 | 0.014 |
|  |  | Simple_repeat | 167,609,661 | 6.652 | 5.535 |
| **Unclassified** |  | Small_RNA | 1,281,401 | 0.051 | 0.042 |
| Low_complexity |  | Unclassified_Other/Composite | 42 | 0 | 0 |
| Simple_repeat |  | Unclassified_RC | 3,269 | 0 | 0 |
| Satellite |  | Unclassified_RC/Helitron | 3,669,354 | 0.146 | 0.121 |
|  |  | Unclassified_RC/Helitron-2 | 68 | 0 | 0 |
|  |  | Unclassified_Retroposon | 195,279 | 0.008 | 0.006 |
|  |  | Unclassified_Unknown | 631,370,054 | 25.056 | 20.849 |
| **Tandem repeats** |  |  | 152,771,249 | 6.058 | 5.045 |
| **Total content** |  |  | 2,521,629,971 | 100 | 83.27% |

## Supplementary Table 20. Statistics of repetitive elements in nine species genomes.

| **Repeat type** | DNA transposon | Non-LTR retroelements (SINES,LINES) | LTR retroelements | Other ( satellites,unknown,low complexity) | Genes and regulatory sequences | Reperts % assembled genome | Genome size (Mb) |
| --- | --- | --- | --- | --- | --- | --- | --- |
| ***S. tuberosum*** | 3.94% | 2.89% | 29.44% | 27.82% | 35.91% | 62.20% | 727 |
| ***S. lycopersicum*** | 0.86% | 0.55% | 61.77% | 4.26% | 36.82% | 68.00% | 828 |
| ***S. melongena*** | 0.85% | 1.63% | 65.80% | 2.96% | 28.76% | 69.08% | 1,170 |
| ***P. floridana*** | 0.71% | 1.54% | 65.69% | 17.94% | 17.76% | 81.59% | 1,389 |
| ***C. annuum*** | 0.47% | 1.18% | 57.25% | 10.72% | 30.38% | 76.36% | 3,061 |
| ***P. tangutica*** | 2.08% | 1.86% | 65.11% | 13.86% | 17.09% | 83.27% | 3,028 |
| ***L. chinense*** | 6.49% | 3.51% | 44.48% | 18.62% | 26.91% | 70.22% | 1,516 |
| ***N. tabacum*** | 2.30% | 1.50% | 58.00% | 8.60% | 29.60% | 78.90% | 4,412 |
| ***Pe. axillaris*** | 5.20% | 2.30% | 40.40% | 15.10% | 36.90% | 65.30% | 1,260 |

|  | Species | Solo-LTR | Paired-LTR | Solo/Paired | Solo-LTR(%) |
| --- | --- | --- | --- | --- | --- |
| *Copia* | ***S. lycopersicum*** | 3,218 | 1,088 | 2.957720588 | 0.747329308 |
|  | ***Ph. floridana*** | 4,866 | 1,159 | 4.198446937 | 0.807634855 |
|  | ***P. tangutica*** | 11,901 | 7,581 | 1.569845667 | 0.610871574 |
|  | ***N. tabacum*** | 1,620 | 1,030 | 1.572815534 | 0.611320755 |
|  | ***L. chinense*** | 1,565 | 2,005 | 0.780548628 | 0.43837535 |
|  | ***Pe. axillaris*** | 3,250 | 2,896 | 1.122237569 | 0.528799219 |
|  | ***C. annuum*** | 4,295 | 1,674 | 2.565710872 | 0.719551014 |
|  | ***S. tuberosum*** | 1,340 | 1,319 | 1.015921152 | 0.503948853 |
|  | ***S. melongena*** | 3,803 | 2,015 | 1.887344913 | 0.653661052 |
| *Gypsy* | ***S. lycopersicum*** | 5,251 | 3,089 | 1.699902881 | 0.629616307 |
|  | ***Ph. floridana*** | 40,995 | 3,909 | 10.48733691 | 0.912947622 |
|  | ***P. tangutica*** | 16,813 | 10,864 | 1.547588365 | 0.607471908 |
|  | ***N. tabacum*** | 14,928 | 5,809 | 2.569805474 | 0.719872691 |
|  | ***L. chinense*** | 7,165 | 1,692 | 4.23463357 | 0.808964661 |
|  | ***Pe. axillaris*** | 9,516 | 6,993 | 1.360789361 | 0.576412866 |
|  | ***C. annuum*** | 11,653 | 4,230 | 2.754846336 | 0.733677517 |
|  | ***S. tuberosum*** | 9,161 | 2,503 | 3.66000799 | 0.785408093 |
|  | ***S. melongena*** | 6,379 | 2,960 | 2.155067568 | 0.683049577 |

## Supplementary Table 21. Comparison of solo-LTRs and paired-LTRs of *Copia* and *Gypsy* elements in the Solanaceae genomes.

## Supplementary Table 22. Accession numbers of T2 RNase genes used in this study.

| Names | Accession numbers |
| --- | --- |
| *Petunia hybrida* SB2-RNase | AB016523.1 |
| *Petunia hybrida* Sx-RNase | AAA33729.1 |
| *Solanum lycopersicum* RNase1 | XP 004229063.1 |
| *Solanum lycopersicum* RNase2 | NP 001234195.1 |
| *Solanum lycopersicum* RNase3 | NP 001234551.2 |
| *Solanum lycopersicum* RNase4 | XP 004237041.1 |
| *Solanum lycopersicum* RNase5 | XP 004242665.1 |
| *Solanum lycopersicum* RNase6 | NP 001307144.1 |
| *Solanum lycopersicum* RNase7 | XP 010323108.1 |
| *Solanum peruvianum* S22-RNase | BAC00930.1 |
| *Solanum peruvianum* S24-RNase | BAC00932.1 |
| *Solanum peruvianum* S7-RNase | CAA81333.1 |
| *Solanum peruvianum* Sn-RNase | CAA81332. |
| *Solanum peruvianum* S-RNase | AAA77040.1 |
| *Solanum tuberosum* RNase1 | XP 006347247.1 |
| *Solanum tuberosum* RNase2 | XP 006355103.1 |
| *Solanum tuberosum* RNase3 | XP 006355102.1 |
| *Solanum tuberosum* RNase4 | XP 006344267.1 |
| *Solanum tuberosum* RNase6 | XP 006344344.1 |
| *Solanum tuberosum* RNase7 | XP 006367819.1 |
| *Solanum tuberosum* RNase8 | XP 006343700.1 |
| *Solanum tuberosum* RNase9 | XP 015162500.1 |

## Supplementary Table 23. Accession numbers of SLF/FBX sequences used in this study

| Names | Accession numbers |
| --- | --- |
| *Petunia hybrida* S11-SLF1 | BAJ24852.1 |
| *Petunia hybrida* S11-SLF2 | BAJ24855.1 |
| *Petunia hybrida* S11-SLF3 | BAJ24861.1 |
| *Petunia hybrida* S11-SLF4 | BAJ24867.1 |
| *Petunia hybrida* S11-SLF5 | BAJ24873.1 |
| *Petunia hybrida* S11-SLF6 | BAJ24879.1 |
| *Petunia hybrida* S19-SLF1 | AAX11681.1 |
| *Petunia hybrida* S5-FBX | BAJ24882.1 |
| *Petunia hybrida* S5-SLF1 | ADD21615.1 |
| *Petunia hybrida* S5-SLF2 | BAJ24853.1 |
| *Petunia hybrida* S5-SLF3 | BAJ24858.1 |
| *Petunia hybrida* S5-SLF4 | BAJ24864.1 |
| *Petunia hybrida* S5-SLF5 | BAJ24870.1 |
| *Petunia hybrida* S5-SLF6 | BAJ24876.1 |
| *Petunia hybrida* S7-SLF2 | BAJ24854.1 |
| *Petunia hybrida* S7-SLF3 | BAJ24859.1 |
| *Petunia hybrida* S7-SLF4 | BAJ24865.1 |
| *Petunia hybrida* S7-SLF5 | BAJ24871.1 |
| *Petunia hybrida* S7-SLF6 | BAJ24877.1 |
| *Petunia hybrida* S9-SLF1 | ADD21614.1 |
| *Petunia hybrida* S9-SLF3 | BAJ24860.1 |
| *Petunia hybrida* S9-SLF4 | BAJ24866.1 |
| *Petunia hybrida* S9-SLF5 | BAJ24872.1 |
| *Petunia hybrida* S9-SLF6 | BAJ24878.1 |
| *Petunia axillaris* S17-SLF2 | BAQ19054.1 |
| *Petunia axillaris* S17-SLF3 | BAJ24862.1 |
| *Petunia axillaris* S17-SLF4 | BAJ24868.1 |
| *Petunia axillaris* S17-SLF5 | BAJ24874.1 |
| *Petunia axillaris* S17-SLF6 | BAJ24880.1 |
| *Petunia axillaris* S19-SLF2 | BAJ24857.1 |
| *Petunia axillaris* S19-SLF3 | BAJ24863.1 |
| *Petunia axillaris* S19-SLF4 | BAJ24869.1 |
| *Petunia axillaris* S19-SLF5 | BAJ24875.1 |
| Petunia axillaris S19-SLF6 | BAJ24881.1 |
| *Solanum habrochaites* SLF1 | KJ814907 |
| *Solanum habrochaites* SLF11 | KJ814928 |
| *Solanum habrochaites* SLF12 | KJ814929 |
| *Solanum habrochaites* SLF13 | KJ814930 |
| *Solanum habrochaites* SLF17 | KU960916 |
| *Solanum habrochaites* SLF20 | KU960917 |
| *Solanum habrochaites* SLF21 | KU960918 |
| *Solanum habrochaites* SLF22 | KU960919 |
| *Solanum habrochaites* SLF23 | KU960920 |
| *Solanum habrochaites* SLF2 | KJ814908 |
| *Solanum habrochaites* SLF4 | KJ814911 |
| *Solanum habrochaites* SLF5 | KJ814915 |
| *Solanum habrochaites* SLF6 | KJ814919 |
| *Solanum habrochaites* SLF7 | KJ814923 |
| *Solanum habrochaites* SLF9 | KJ814926 |
| *Solanum lycopersicum* SLF1 | KJ814895.1 |
| *Solanum lycopersicum* SLF11 | KJ814900 |
| *Solanum lycopersicum* SLF12 | KJ814901 |
| *Solanum lycopersicum* SLF13 | KJ814902 |
| *Solanum lycopersicum* SLF9 | KJ814898 |
| *Solanum lycopersicum* Chr9_FBX2 | SlySL4.0ch09g2318 |
| *Solanum lycopersicum* Chr9_FBX1 | SlySL4.0ch09g1724 |
| *Solanum lycopersicum* Chr10_FBX1 | SlySL4.0ch10g1332 |
| *Solanum lycopersicum* Chr10_FBX2 | SlySL4.0ch10g1594 |
| *Solanum lycopersicum* Chr5_FBX1 | SlySL4.0ch05g15 |
| *Solanum lycopersicum* Chr7_FBX1 | SlySL4.0ch07g1305 |
| *Solanum habrochaites* S1-SLF2 | AIG62971.1 |
| *Solanum habrochaites* S1-SLF4 | AIG62973.1 |
| *Solanum habrochaites* S1-SLF5 | AIG62980.1 |
| *Solanum habrochaites* S1-SLF6 | AIG62983.1 |
| *Solanum habrochaites* S1-SLF7 | AIG62987.1 |
| *Solanum habrochaites* S1-SLF9 | AIG62937.1 |
| *Solanum habrochaites* S5-SLF11 | AIG62990.1 |
| *Solanum habrochaites* S5-SLF12 | AIG62991.1 |
| *Solanum habrochaites* S5-SLF13 | AIG62992.1 |
| *Solanum habrochaites* S5-SLF2 | AIG62970.1 |
| *Solanum habrochaites* S5-SLF4 | AIG62933.1 |
| *Solanum habrochaites* S5-SLF6 | AIG62981.1 |
| *Solanum habrochaites* S5-SLF7 | AIG62985.1 |

## Supplementary Table 24. Resequenced sample information.

| **ID** | **EffectiveDepth** | **Sample distribution** | **Altitude** | **Accession number** | **Inbreeding coefficient (*F*_IS_ )** | **Selfing rate**  **(*s*)** |
| --- | --- | --- | --- | --- | --- | --- |
| Pop1-1 | 34.337636 | 34°35.57′N; 98°29.00′E | 4074 m | SRR17331579 | 0.8214 | 0.9019 |
| Pop1-2 | 21.305903 | 34°35.57′N; 98°29.00′E | 4074 m | SRR17331578 | 0.7882 | 0.8816 |
| Pop1-3 | 24.347309 | 34°35.57′N; 98°29.00′E | 4074 m | SRR17331577 | 0.7830 | 0.8783 |
| Pop1-4 | 20.799216 | 34°35.57′N; 98°29.00′E | 4074 m | SRR17331575 | 0.8177 | 0.8997 |
| Pop1-5 | 27.111391 | 34°35.57′N; 98°29.00′E | 4074 m | SRR17331576 | 0.7916 | 0.8837 |
| Pop2-1 | 20.708015 | 34°84.17′N; 98°43.82′E | 4181 m | SRR17331592 | 0.7927 | 0.8844 |
| Pop2-2 | 26.598076 | 34°84.17′N; 98°43.82′E | 4181 m | SRR17331591 | 0.7070 | 0.8284 |
| Pop2-3 | 28.841084 | 34°84.17′N; 98°43.82′E | 4181 m | SRR17331580 | 0.8299 | 0.9070 |
| Pop2-4 | 28.771534 | 34°84.17′N; 98°43.82′E | 4181 m | SRR17331569 | 0.8397 | 0.9129 |
| Pop2-5 | 21.934146 | 34°84.17′N; 98°43.82′E | 4181 m | SRR17331568 | 0.6969 | 0.8214 |
| Pop3-1 | 19.633544 | 34°23.75′N; 95°77.56′E | 4377 m | SRR17331567 | 0.1798 | 0.3048 |
| Pop3-2 | 23.416251 | 34°23.75′N; 95°77.56′E | 4377 m | SRR17331566 | 0.1693 | 0.2896 |
| Pop3-3 | 19.350457 | 34°23.75′N; 95°77.56′E | 4377 m | SRR17331565 | 0.1794 | 0.3042 |
| Pop3-4 | 24.307881 | 34°23.75′N; 95°77.56′E | 4377 m | SRR17331564 | 0.1892 | 0.3182 |
| Pop3-5 | 26.720233 | 34°23.75′N; 95°77.56′E | 4377 m | SRR17331563 | 0.1713 | 0.2925 |
| Pop4-1 | 23.963186 | 33°05.47′N; 96°85.46′E | 3987 m | SRR17331590 | 0.0653 | 0.1226 |
| Pop4-2 | 25.363900 | 33°05.47′N; 96°85.46′E | 3987 m | SRR17331589 | 0.0559 | 0.1059 |
| Pop4-3 | 28.252018 | 33°05.47′N; 96°85.46′E | 3987 m | SRR17331588 | 0.0466 | 0.0891 |
| Pop4-4 | 22.875283 | 33°05.47′N; 96°85.46′E | 3987 m | SRR17331587 | 0.0454 | 0.0869 |
| Pop4-5 | 21.990103 | 33°05.47′N; 96°85.46′E | 3987 m | SRR17331586 | 0.0496 | 0.0945 |
| Pop5-1 | 18.696996 | 33°57.17′N; 92°06.78′E | 4681 m | SRR17331585 | 0.1207 | 0.2154 |
| Pop5-2 | 21.375733 | 33°57.17′N; 92°06.78′E | 4681 m | SRR17331584 | 0.0423 | 0.0812 |
| Pop5-3 | 23.951517 | 33°57.17′N; 92°06.78′E | 4681 m | SRR17331583 | 0.0286 | 0.0556 |
| Pop5-4 | 18.060618 | 33°57.17′N; 92°06.78′E | 4681 m | SRR17331582 | 0.0005 | 0.0010 |
| Pop5-5 | 21.884111 | 33°57.17′N; 92°06.78′E | 4681 m | SRR17331581 | 0.0125 | 0.0247 |
| Pop6-1 | 18.539164 | 31°36.47′N; 93°07.40′E | 4099 m | SRR17331574 | 0.0553 | 0.1048 |
| Pop6-2 | 25.341809 | 31°36.47′N; 93°07.40′E | 4099 m | SRR17331573 | 0.1188 | 0.2124 |
| Pop6-3 | 18.525465 | 31°36.47′N; 93°07.40′E | 4099 m | SRR17331572 | 0.0810 | 0.1499 |
| Pop6-4 | 18.617193 | 31°36.47′N; 93°07.40′E | 4099 m | SRR17331571 | 0.0711 | 0.1328 |
| Pop6-5 | 18.843336 | 31°36.47′N; 93°07.40′E | 4099 m | SRR17331570 | 0.0752 | 0.1399 |

## Supplementary Table 25. HyDe results of hybridization detection analyses using the run_hyde.py script.

| P1 | Hybrid | P2 | Z-score | P value ^a^ | γ^b^ |
| --- | --- | --- | --- | --- | --- |
| East | Central_pop4 | Central_pop3 | -974.725 | ~1.0 | 0.575 |
| East | Central_pop3 | Central_pop4 | 345.981 | ~0.0 | 0.793 |
| Central_pop3 | East | Central_pop4 | -99999.9 | ~1.0 | -0.563 |

Note: P1 and P2 correspond to two putative parental populations respectively, and Hybrid was the potential hybrid population. a. P value less than 0.05 indicates a significant signal of hybridization event. b. γ parameter is the probability that Hybrid is sister to P2. In the hybrid speciation model, it represents the relative proportion of the genetic composition derived from P2.

## Supplementary Table 26. Genomic diversity (π) of three lineages*.*

| lineage | observed_π |
| --- | --- |
| Central | 0.00108 |
| East | 0.000131 |
| West | 0.000871 |
| total | 0.00122 |
